# Scaling Theory of a Polymer Ejecting from a Cavity into a Semi-Space

**DOI:** 10.3390/polym12123014

**Published:** 2020-12-16

**Authors:** Pai-Yi Hsiao

**Affiliations:** 1 Department of Engineering and System Science, National Tsing Hua University, Hsinchu 300044, Taiwan; pyhsiao@ess.nthu.edu.tw or pyhsiao@mx.nthu.edu.tw; Tel.: +886-3-516-2247; 2 Institute of Nuclear Engineering and Science, National Tsing Hua University, Hsinchu 300044, Taiwan

**Keywords:** polymer ejection, scaling theory, molecular dynamics simulations

## Abstract

A two-stage model is developed in order to understand the scaling behaviors of single polymers ejecting from a spherical cavity through a nanopore. The dynamics of ejection is derived by balancing the free energy change with the energy dissipation during a process. The ejection velocity is found to vary with the number of monomers in the cavity, *m*, as $m^{z_{1}}/\left(N^{x_{1}}D^{3z_{1}}\right)$ at the confined stage, and it turns to be $m^{-z_{2}}$ at the non-confined stage, where *N* is the chain length and *D* the cavity diameter. The exponents are shown to be $z_{1}={\left(3\nu -1\right)}^{-1}$, $z_{2}=2\nu$ and $x_{1}=1/3$, with ν being the Flory exponent. The profile of the velocity is carefully verified by performing Langevin dynamics simulations. The simulations further reveal that, at the starting point, the decreasing of *m* can be stalled for a good moment. It suggests the existence of a pre-stage that can be explained by using the concept of a classical nucleation theory. By trimming the pre-stage, the ejection time are properly studied by varying *N*, *D*, and $\phi_{0}$ (the initial volume fraction). The scaling properties of the nucleation time are also analyzed. The results fully support the predictions of the theory. The physical pictures are given for various ejection conditions that cover the entire parameter space.

## 1. Introduction

The ejection of polymer is a process concerning a polymer being ejected from a confined space into a non-confined space through a small pore. A well-known example can be found in a bacteriophage, a virus that is able to infect a bacterial cell by injecting its genetic materials that are encapsulated in the capsid head into the cell through a channel tail [1,2]. During the process, it is noticed that the capsid does not change the volume. In mimicking the nature, researchers have developed various new techniques in order to manipulate genetic molecules, for example, trapping DNA molecules in a cage and ejecting it through a pore [3], flossing a DNA chain between two nanocages [4], transporting DNA into a double barrel nanopore device [5], and encapsulating genome molecules in engineered protein cages for gene therapy [6]. For the successful development of these techniques, a fundamental understanding of the packaging and ejecting mechanisms of biopolymers into and from a closed shell is necessary.

For years, continuum mechanics models have been used in order to explain DNA ejection [2,7,8,9,10,11,12,13]. In this type of models, a DNA molecule is regarded as an elastic string that is packaged in a virion capsid. The osmotic pressure in the virion is much higher than the one outside because of various factors, such as the high packing fraction of the DNA chain in the virion, the large bending energy in arranging the chain in the small space of the capsid, the strong electrostatic repulsion between the strands of the packaged chain, and so on. The pressure difference is the driving force that pushes the chain out of the capsid. However, the internal pressure decreases with advancing of the process. The ejection shall be inhibited when the pressure drops to a value that is balanced with the external one [11,13,14]. Therefore, the models predict a full ejection timescale that is much longer than what has been observed in experiments [1]. To solve the problem, a second mechanism was proposed, in which the ejected DNA chain binds with some proteins in the cytoplasm [9]. The binding reduces the energy of the system and, consequently, pulls the rest of the chain into the cell [10,12]. Recently, a hydrodynamic type of models has been suggested, where the penetration of water molecules across the capsid shell is considered [15,16,17,18]. It is thought that the inward flow of water provides the necessary driving to flush the chain out of the virion through the tail channel. Moreover, a condensed DNA molecule in the capsid is dehydrated. The rehydration of the DNA chain, when it leaves the capsid, helps in the completion of the ejection.

Scaling analysis in the domain of polymer physics provides an alternative approach for understanding the phenomena. Muthukumar used the Fokker–Planck equation and the classical nucleation theory to study polymer ejection from a cavity [19,20,21]. The ejection time $\tau ∼N^{1+(1/3\nu )}\phi_{0}^{-1/3\nu }$ was predicted, where *N* is the chain length, $\phi_{0}$ is the initial packing fraction of the chain in the cavity, and ν is the Flory exponent [22]. Cacciuto and Luijten combined the scaling bound for translocation time $\tau ∼N^{1+\nu }/\Delta \mu$ [23] with the free energy cost of confining $\Delta F∼N\phi_{0}^{1/(3\nu -1)}$, and argued that the ejection time should scale as $N^{1+\nu }\phi_{0}^{-1/(3\nu -1)}$ [24,25]. Sakaue and Yoshinaga noticed that the chemical potential gradient Δμ should gradually decrease as the process advanced [26]. The dynamics of ejection were studied by equating the free energy change with the energy dissipation in the proximity of the pore within a correlation length ξ(t). The ejection time was deduced to be $\tau ∼N^{\left(2+\nu )/(3\nu \right)}\phi_{0}^{-(2+\nu )/(3\nu )}$ in the osmotic-driven regime.

Simulations have been largely invested in the study of polymer ejection and translocation [27,28,29]. It was found that the ejection process evolves faster for an orderly packed DNA spool than a disordered or knotted DNA chain [30,31]. The geometry of capsid plays a non-trivial role in the determination of the dynamics of ejection [32,33,34,35]. Truncating the tail channel of the virion or increasing the solvent temperature accelerates the evolution of ejection [36]. Other effects that are able to affect the ejection include the chain rigidity [34,37,38,39], the solvent quality [40,41], the hydrodynamics [36,42], the electrostatics [36,43], the pore dimension [44,45,46], and so on. These effects have been the main topics of investigation in the past two decades. The simulations also calculated the free energy landscape [33,34] that allow researchers to understand the variation of the thermodynamic state during the ejection process. Despite the influence of the above factors and the complexity of the results, the reported ejection behaviors display many similarities in various aspects. Thus, it is believed that there exists a universal mechanism that fundamentally controls the evolution of an ejection.

In order to verify the conjecture, we have recently developed a scaling theory to explain polymer ejection from a cavity through a small pore [47]. The central idea used is to balance the free energy change with the energy dissipated as the chain passes the pore. The dynamics of ejection was studied in the confined and non-confined stages separately. We were able to solve the two dynamical equations and the ejection time was found to be $\tau ∼N^{\left(2+\nu )/(3\nu \right)}\phi_{0}^{-2/(3\nu )}$. Molecular dynamics simulations were then performed and the results support the prediction of our theory. However, there are still various scaling concepts and behaviors to be verified. Is our scaling theory a general and consistent theory that is able to pass the stringent numerical examinations under broad and different simulation conditions? What kinds of scaling pictures are we waiting for from a primitive model where a simple bead-spring chain is ejected out of a spherical cavity? Only with a good knowledge of the primitive model we are able to go further to assess the impacts that are brought in by the other effects, such as the chain stiffness, hydrodynamics, electrostatics, etc., in order to gain a better understanding of a real ejection process that happened in nature and the applications.

In this work, the details of the derivation of our scaling theory will be provided (Section 2). The prediction will be then verified by performing elaborated molecular simulations described in Section 3. The results of simulation will be reported and discussed in Section 4. The studied topics include the scaling of the ejection velocity (Section 4.1) and the time evolution of the number of monomers in a cavity (Section 4.2). We will show that an ejection process can be truly distinguished into the confined and non-confined stages. We further show that, prior to the confined stage, the change of the number of monomers in the cavity can be stalled for a long while, depending on the channel length. The phenomena can be explained by using the concept of the nucleation theory. The scaling behaviors for the nucleation time and the following ejection time will be properly investigated by systematically varying the chain length, the cavity size, and the initial packing fraction (Section 4.3). Section 5 will provide the overall discussions and conclusions.

## 2. Scaling Theory of Ejection Dynamics

We deal with the problem of a polymer ejected from a spherical cavity, through a small pore, to an open semi-space. The chain comprises *N* monomers, which are represented by *N* beads. The bead diameter is σ and the length of the connecting bonds is *b*. For simplicity, we assume that b=σ. The diameter of the cavity is *D*. Therefore, the initial volume fraction of monomers in the cavity is $\phi_{0}=N{\left(\sigma /D\right)}^{3}$. The head monomer is positioned at the pore entrance. In order to guarantee the success of an ejection, there exists some mechanism at the pore entrance that prevents the falling of the head monomer into the cavity.

An ejection process can be subdivided into two stages, which are demarcated by the overlap volume fraction $\phi_{*}$ of the space. It can be shown that $\phi_{*}$ scales as ${\left(\sigma /D\right)}^{1/(z_{1}\nu )}$ with $z_{1}=1/\left(3\nu -1\right)$ for the cavity. At the first stage, the volume fraction $\phi =m{\left(\sigma /D\right)}^{3}$ is higher than $\phi_{*}$, where *m* denotes the number of the monomers in the cavity at the instant. Thus, the chain “feels” the restriction of the confinement and it is “pressed” to go outside through the pore. We call it the confined stage. The second stage is called the non-confined stage, which takes place when $\phi <\phi_{*}$, or, equivalently, when *m* becomes smaller than the critical value $m_{*}∼{\left(D/\sigma \right)}^{1/\nu }$. At this stage, the internal monomers do not feel the pressing of the confinement. They are driven out of the cavity by entropic pulling of the external chain segments [48]. We remark that, for the case of the ejection starting with a short chain $N<N_{*}$, or, equivalently, $\phi_{0}<\phi_{*}$, the process will only be proceeded via the non-confined stage, because the chain size has been smaller than the cavity size since the beginning. Here, $N_{*}$ is the critical chain length for the occurrence of a two-stage process and it scales as ${\left(D/\sigma \right)}^{1/\nu }$ [49].

At the confined stage $m\geq m_{*}$, the free energy *F* of the chain in the cavity can be calculated from the blob theory. Each blob has a free energy $k_{B}T$ and, thus, $F∼k_{B}Tm/g$. Here, *T* is the temperature, $k_{B}$ is the Boltzmann constant, and *g* is the number of the monomers in a blob. By equating the volume fraction in a blob $\phi_{b}$ to the instantaneous volume fraction ϕ, we have $g∼\phi^{-z_{1}}∼m^{-z_{1}}m_{*}^{3\nu z_{1}}$ and, thus, $F∼k_{B}T{\left(m/m_{*}\right)}^{3\nu z_{1}}$. The dynamics of ejection can be studied by balancing the rate of the free energy change, dF/dt, with the rate of the energy dissipation occurred at the pore, $-\eta V_{\mathrm{ej}}^{2}$, where η is the effective friction coefficient and $V_{\mathrm{ej}}=-\sigma \left(dm/dt\right)$ is the ejection velocity. It yields

(1) $$\frac{dm}{dt}∼-\frac{k_{B}T}{\eta \sigma^{2}}\frac{d(m/g)}{dm}∼\frac{-1}{\Delta t}{\left(\frac{m}{m_{*}^{3\nu }}\right)}^{z_{1}}$$

where $\Delta t=\eta \sigma^{2}/k_{B}T$ is the characteristic time. We will show later in the simulations that Δt exhibits a scaling dependence on *N* as $\Delta t∼N^{x_{1}}\Delta t_{0}$. This additional dependence can be attributed to the change of the friction coefficient under the form $\eta ∼\eta_{0}N^{x_{1}}$; here, $\eta_{0}$ is the friction coefficient of the solvent. A number of the effects could cause the results, for example, the connectivity of a chain, the geometrical restriction to transport chain segments from the cavity to the pore channel, the jamming of the monomers accumulated outside the pore, which hinders the progress of ejection, and so on.

Solving the differential equation with the two conditions: (1) m=N at t=0, (2) $m=m_{*}$ at $t=\tau_{1}$, we obtain the ejection time for the first stage

(2) $$\tau_{1}∼\frac{\Delta t}{z_{1}-1}\left[m_{*}^{2}-m_{*}^{1+z_{1}}N^{1-z_{1}}\right]∼\frac{\Delta t_{0}N^{x_{1}}}{z_{1}-1}m_{*}^{2}\left[1-{\left(\frac{m_{*}}{N}\right)}^{z_{1}-1}\right].$$

Thus, the decrease of the number of monomers with time is predicted by

(3) $$m≃N{\left(1+\frac{t}{t_{0}}\right)}^{-\zeta_{1}}$$

where $t_{0}≃\zeta_{1}m_{*}^{1+z_{1}}N^{1-z_{1}}\Delta t$ and $\zeta_{1}=1/\left(z_{1}-1\right)$.

When $m<m_{*}$, the process is at the non-confined stage. The free energy is approximately $F∼k_{B}T\left[\left(1-\gamma_{i}^{\prime }\right)\ln m+\left(1-\gamma_{o}^{\prime }\right)\ln\left(N-m\right))\right]-m\Delta \mu_{io}$, where $\gamma_{i}^{\prime }$ and $\gamma_{o}^{\prime }$ are the exponents for the scaling of the partition function of a chain tethered inside and outside of the cavity, respectively, and $\Delta \mu_{io}$ is the chemical potential difference [19,21,22]. Equating the rate of the free energy change to the rate of the energy dissipation, we have

(4) $$\frac{dm}{dt}∼\frac{-1}{\Delta t}\left[\frac{1-\gamma_{i}^{\prime }}{m}-\frac{1-\gamma_{o}^{\prime }}{N-m}-\frac{\Delta \mu_{io}}{k_{B}T}\right].$$

In the long chain limit, $N\gg m_{*}$, the second term on the right-hand side is much smaller than the first term and, thus, can be neglected. The third term can also be ignored because *m* is small at the non-confined stage. Consequently, the dynamics of ejection is mainly determined by the scaling equation

(5) $$\frac{dm}{dt}∼\frac{-1}{\Delta t}m^{-1}∼\frac{-1}{\Delta t_{0}}m^{-z_{2}}.$$

The simulations shown later suggest that η should scale as $\eta_{0}m^{y_{2}}$. The effect enters the problem through $\Delta t=m^{y2}\Delta t_{0}$. Hence, the dynamics has a $m^{-z_{2}}$ scaling dependence with $z_{2}=1+y_{2}$. The ejection time for the non-confined stage is obtained by solving Equation (5) with the boundary conditions: (1) $m=m_{*}$ at $t=\tau_{1}$, (2) m=0 at $t=\tau_{1}+\tau_{2}$, and reads as

(6) $$\tau_{2}∼\frac{\Delta t_{0}}{1+z_{2}}m_{*}^{1+z_{2}}$$

The time variation of *m* before the ending of ejection is predicted by

(7) $$m≃M_{0}{\left(1-\frac{t}{\tau_{\mathrm{ej}}}\right)}^{\zeta_{2}}$$

where $M_{0}={\left(\frac{z_{2}+1}{\Delta t_{0}}\tau_{\mathrm{ej}}\right)}^{\zeta_{2}}$, $\zeta_{2}=1/\left(z_{2}+1\right)$, and $\tau_{\mathrm{ej}}=\tau_{1}+\tau_{2}$ is the total ejection time.

We comment that, in the ejection problem, the condition of a process is controlled by the three main parameters: the chain length *N*, the cavity diameter *D*, and the initial volume fraction $\phi_{0}$. These parameters are not totally independent, because $\phi_{0}=N{\left(\sigma /D\right)}^{3}$. Thus, the ejection time can be expressed by using any two of the three parameters. Table 1 provides three ways to express $\tau_{\mathrm{ej}}$, where $A_{1}$ and $A_{2}$ are the scaling prefactors for the two stages, respectively [50].

The $\tau_{\mathrm{ej}}$ function is divided into two pieces: one is applied for the ejection simply proceeded via the non-confined stage, happened for small *N*, small $\phi_{0}$, or large *D*, and the other is applied for a typical ejection experiencing the confined and then the non-confined stage. The two columns on the right side of the table give the two relevant ways to regard the ejection time by fixing one of the two parameters. For example, the second column on the right side of the table for formula (a) indicates that $\tau_{\mathrm{ej}}$ is studied under the *D*-fixed condition. The expression is demarcated by the the critical value $N_{*}∼{\left(D/\sigma \right)}^{1/\nu }$ and the two pieces of function are applied for the situations $N<N_{*}$ and $N\geq N_{*}$, respectively.

**Figure 1:**
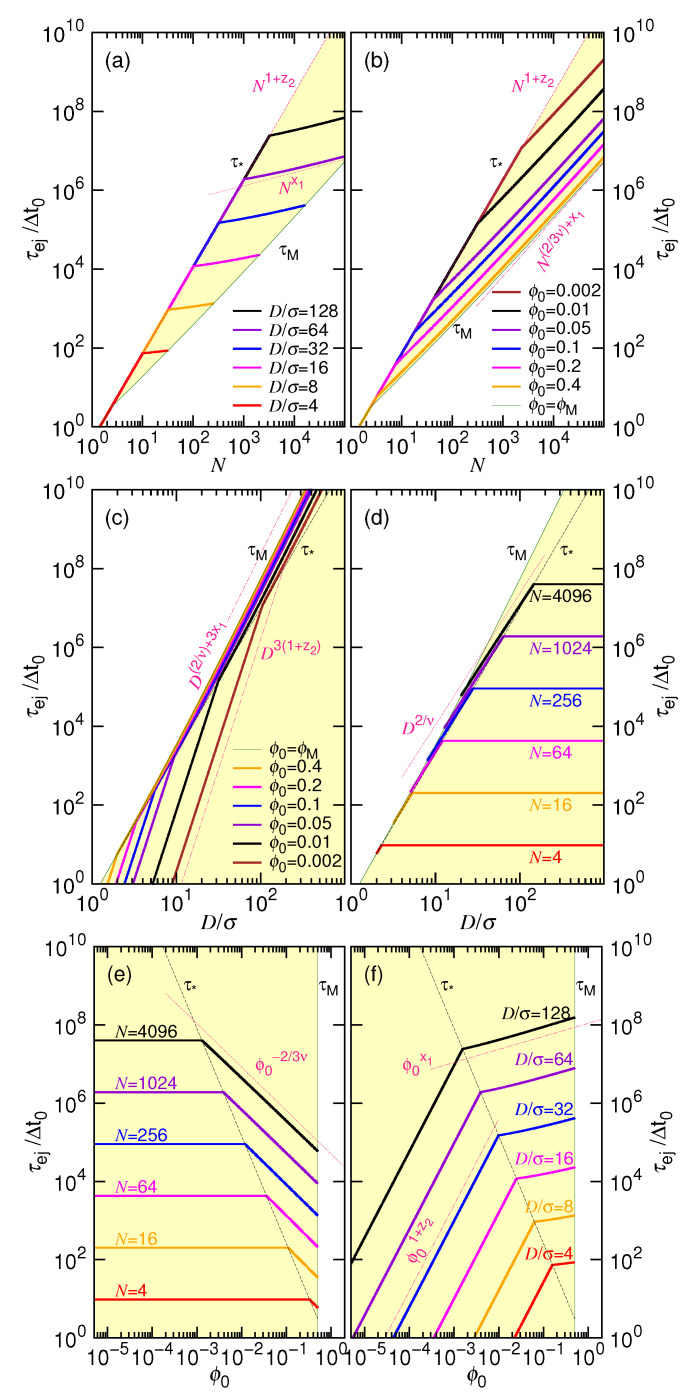
(**a**) Ejection time $\tau_{\mathrm{ej}}$ vs. chain length *N* at fixed cavity diameter *D*; (**b**) $\tau_{\mathrm{ej}}$ vs. *N* at fixed initial volume fraction $\phi_{0}$; (**c**) $\tau_{\mathrm{ej}}$ vs. *D* at fixed $\phi_{0}$; (**d**) $\tau_{\mathrm{ej}}$ vs. *D* at fixed *N*; (**e**) $\tau_{\mathrm{ej}}$ vs. $\phi_{0}$ at fixed *N*; (**f**) $\tau_{\mathrm{ej}}$ vs. $\phi_{0}$ at fixed *D*. The plots are made by setting ν=0.6, $x_{1}=1/3$, $y_{2}=0.2$, $A_{1}=0.04$, and $A_{2}=1.0$. The $\tau_{*}$ curve describes the ejection time occurred at the critical value. The $\tau_{M}$ curve presents the ejection time at the maximum allowed $\phi_{0}$, assumed to be $\phi_{M}=0.5$. Important scaling behaviors are indicated in the plots by using dark-pink dashed or dotted lines.

To see the variation, we make the plots of the ejection time in Figure 1.

Panels (a) and (b) present $\tau_{\mathrm{ej}}$ vs. *N* under the *D*-fixed and $\phi_{0}$-fixed conditions, respectively. Similarly, Panels (c) and (d) are the plots of $\tau_{\mathrm{ej}}$ vs. *D* at fixed $\phi_{0}$ and fixed *N*; Panels (e) and (f) are the time plot against $\phi_{0}$ at different values of *N* and *D*. The plots are made by setting ν=0.6, $x_{1}=1/3$, $y_{2}=0.2$, $A_{1}=0.04$, and $A_{2}=1.0$. We assume that the maximum allowed value of $\phi_{0}$ is 0.5, being denoted by $\phi_{M}$, which defines the ejection time boundary $\tau_{M}$ in each plot. The $\tau_{*}$ curve shows the ejection time at the critical point, either at $\phi_{0}=\phi_{*}$, at $N=N_{*}$, or at $D=D_{*}$. The yellow region on the plots indicates the codomain of the $\tau_{\mathrm{ej}}$ function when a concerned parameter, *N*, *D*, or $\phi_{0}$, is varied. A $\tau_{\mathrm{ej}}$ curve in the codomain shows how the ejection time varies at a given value of parameter.

The asymptotic scaling behaviors are drawn on the plots in dark-pink dashed or dotted lines. In the long chain limit (refer to Panels (a) and (b)), the ejection time scales as $N^{x_{1}}$ at a given *D* and as $N^{2/\left(3\nu \right)+x_{1}}$ at a given $\phi_{0}$. If the process solely lies at the non-confined stage, the predicted scaling is $N^{1+z_{2}}$. Panel (c) reveals that the scaling is $D^{3(1+z_{2})}$ when $D\leq D_{*}$ and $D^{\left(2/\nu \right)+3x_{1}}$ when $D>D_{*}$. If it is *N* being fixed (see Panel (d)), $\tau_{\mathrm{ej}}$ scales as $D^{2/\nu }$ for the situation pressed by the confinement. Concerning the variation with $\phi_{0}$, a scaling decrease is expected in Panel (e) in the large $\phi_{0}$ region, with the exponent being equal to $-\frac{2}{3\nu }$. If *D* is fixed (refer to Panel (f)), then the exponent is $x_{1}$ for $\phi_{0}\geq \phi_{*}$ and $1+z_{2}$ for $\phi_{0}<\phi_{*}$.

**Figure 2:**
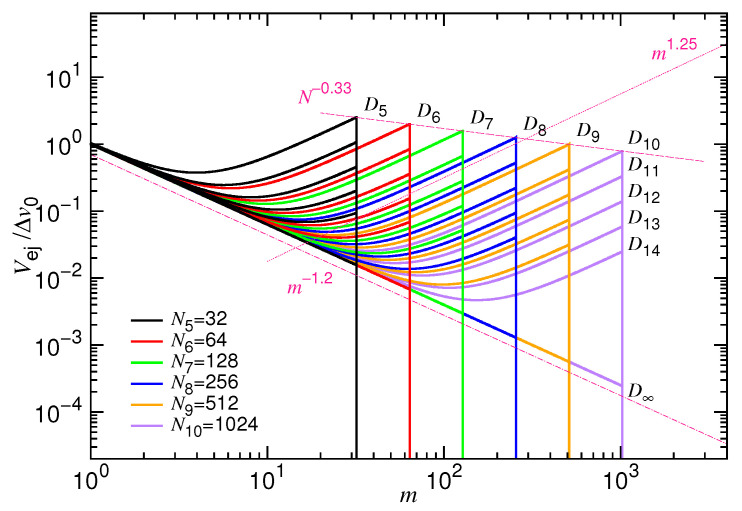
Ejection velocity $V_{\mathrm{ej}}$ as a function of *m* in an ejection process. The plots are made by setting $z_{1}=1.25$, $z_{2}=1.2$, $x_{1}=1/3$, $A_{1}=0.04$, and $A_{2}=1$. Different chain lengths $N_{i}=2^{i}$ are studied and given in the legend. The cavity diameter *D* is indicated near the right-top side of the branch curves, and varied from $D_{5}=\sqrt[{3}]{2.5\times 2^{5}}\sigma$ to $D_{14}=\sqrt[{3}]{2.5\times 2^{14}}\sigma$. The velocity curves for the infinite diameter $D_{\infty }$ are plotted as references.

The ejection velocity in a process can be studied by combining Equation (1) and Equation (5) into one equation

(8) $$V_{\mathrm{ej}}∼\Delta v_{0}\left[\frac{1}{A_{1}N^{x_{1}}}{\left(\frac{m}{{\left(D/\sigma \right)}^{3}}\right)}^{z_{1}}+\frac{1}{A_{2}}m^{-z_{2}}\right]$$

where $\Delta v_{0}\equiv \sigma /\Delta t_{0}$ is the characteristic velocity, and $A_{1}$ and $A_{2}$ are the two prefactors [50]. Figure 2 presents the predicted $V_{\mathrm{ej}}$ vs. *m* at different *N* and *D* values. The curves are plotted using the same set of setting as Figure 1, which gives $z_{1}=1.25$ and $z_{2}=1.2$. Because the instant number *m* of monomers in the cavity decreases with time, the curves should be read from the right to the left, in order to follow the direction of time evolution.

We can see that the combined equation, Equation (8), preserves the required scaling behavior $m^{z_{1}}$ at the confined stage and $m^{-z_{2}}$ at the non-confined stage. The turning point between the two scaling behaviors defines the demarcating monomer number $m_{*}$. Noticeably, the departure velocity of ejection at m=N exhibits an apparent decreasing behavior $N^{-0.33}$ with the chain length at a fixed $\phi_{0}$ value, as indicated in the figure by a dark-pink dashed line. It results from the $N^{x_{1}}$ term that is given in the denominator of the equation. We will come back to this topic later. It is quite amazing to discover that, despite the different departure conditions, the velocity curves all evolve to converge and, finally, follow the ones for the cases with an infinitely large diameter.

The ejection time and ejection velocity will be systematically studied later in this paper by means of molecular dynamics simulations. Analysis will be performed in order to verify all of the details of the scaling behaviors.

## 3. Simulation Model and Setup

**Figure 3:**
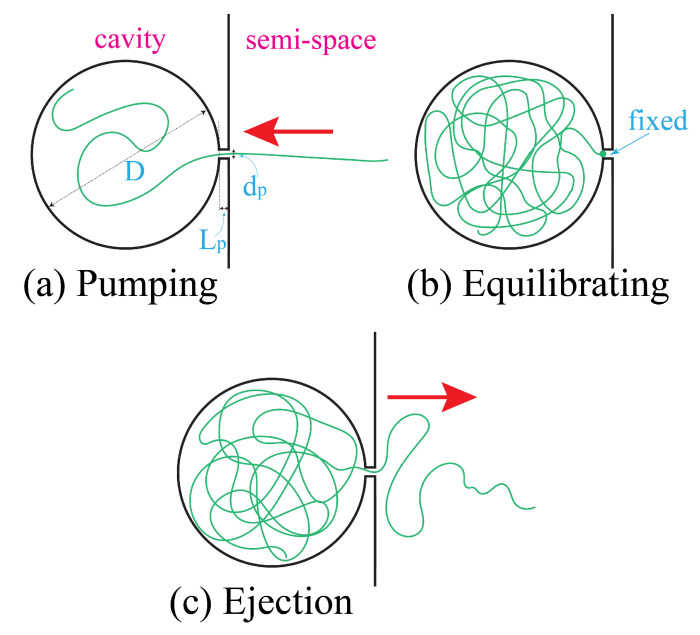
Sketches of the three processing steps in a simulation: (**a**) pumping, (**b**) equilibrating, and (**c**) ejection. *D* and $d_{p}$ denote the effective diameters of the cavity and the pore, respectively. $L_{p}$ is the length of the pore. The green line represent the chain. The red arrow indicates the direction of transportation of the chain.

We performed molecular dynamics simulations to examine the ejection theory of a polymer. The polymer is modeled as a bead-spring chain and pumped into a spherical cavity for confinement. The confined chain is then equilibrated under a constraint by attaching the chain end (i.e., the head monomer) at the pore entrance, which blocks the exit of the chain. In order to start an ejection, the constraint is removed, and the chain ejects out of the cavity through the pore spontaneously. The three processing steps: pumping, equilibrating, and ejection, are sketched in Figure 3.

Each bead on the chain represents a monomer. The Weeks–Chandler–Andersen potential models the excluded volume interaction between beads [51],

$$U_{\mathrm{ex}}\left(r\right)=\left\{\begin{array}{cc} 4\varepsilon \left[{\left(\frac{\sigma }{r}\right)}^{12}-{\left(\frac{\sigma }{r}\right)}^{6}\right]+\varepsilon & \mathrm{for}\;r\leq \sqrt[{6}]{2}\sigma \\ 0 & \mathrm{for}\;r>\sqrt[{6}]{2}\sigma \end{array}\right.$$

which is a Lennard–Jones (LJ) 12–6 potential, truncated and shifted at the minimum point, where *r* is the distance between two beads, and ε and σ are the interaction strength and length of the LJ potential, respectively. The bonding between two monomers is modeled by a harmonic potential and read as $U_{\mathrm{bd}}\left(b\right)=\frac{1}{2}k{\left(b-b_{0}\right)}^{2}$, where *k* is the spring constant and $b-b_{0}$ is the stretching length of a bond. The beads interact with the cavity wall via a LJ 9–3 potential, $U_{w}\left(r\right)=\varepsilon_{w}\left[\frac{2}{15}{\left(\frac{\sigma_{w}}{r}\right)}^{9}-{\left(\frac{\sigma_{w}}{r}\right)}^{3}\right]$, truncated at $r=\sqrt[{6}]{\frac{2}{5}}\sigma_{w}$. We set $\varepsilon_{w}=3\varepsilon$, $\sigma_{w}=\sigma$, $k=600\varepsilon /\sigma^{2}$, and $b_{0}=\sigma$. The thermal fluctuations are modeled using the Langevin thermostat with the desired temperature set at $T=1.0\;\varepsilon /k_{B}$ and the damping time set to $t_{D}=1.0\;\sigma \sqrt{m/\varepsilon }$ [52]. Here, $k_{B}$ is the Boltzmann constant and m is the mass of a bead. Under this setting, the LJ 9–3 wall potential attains an energy that is equal to the thermal energy $k_{B}T$ at r≃0.75σ. It defines the thickness of the wall to be 0.25σ. Therefore, in order to simulate ejection from a cavity of diameter *D*, we have to set the wall on a sphere of diameter equal to $D_{C}=D+0.5\sigma$, centered at the cavity center. A pore is opened on the wall in order to connect the inner cavity space with the outer semi-space. It is modeled by a cylinder while using the same wall potential. The pore diameter is effectively $d_{p}=1.5\sigma$ and the pore length is $L_{p}=1.0\sigma$.

The ejection process is investigated by varying the number of beads on a chain, *N*, and the diameter of the cavity, *D*. In order to study the scaling behavior, we change *N* systematically from 16 to 1024, as a power of 2, and denote it by $N_{g_{N}}=2^{g_{N}}$. The diameter *D* is set in order to produce a desired initial volume fraction of monomers in the cavity, $\phi_{0}$, at a given *N* value. The highest $\phi_{0}$ studied is set to 0.4, because a typical volume fraction of DNA in a bacteriophage is around the value [2,11]. We decrease the value of $\phi_{0}$ by half for each time, in order to generate a series of the studied cases at the initial volume fraction equal to $0.4\times 2^{-g_{F}}$, denoted by $\phi_{0,g_{F}}$. Thus, the diameter *D* is equal to ${\left(2.5\times 2^{g_{N}+g_{F}}\right)}^{1/3}\sigma$, because of $\phi_{0}=N\sigma^{3}/D^{3}$. We denote it by $D=D_{g_{D}}$, and the relation between the three generation numbers is $g_{D}=g_{N}+g_{F}$. The organization of these studying cases allows for us to investigate scaling behaviors in a logical way under different combinations of the parameters. Five hundred independent runs are performed for each studied ($N_{g_{N}}$, $D_{g_{D}}$) case. The simulation trajectories are recorded and analyzed using standard statistical methods.

In the following text, the quantities m, σ, and ε will be used as the mass, the length, and the energy unit, respectively, in order to describe data. A physical quantity will be reported by only giving the value without mentioning the unit. For example, the ejection time “$\tau_{\mathrm{ej}}=80.0$” means $\tau_{\mathrm{ej}}=80.0\;t_{u}$, where $t_{u}=\sigma \sqrt{m/\varepsilon }$ is the time unit. The velocity “$V_{\mathrm{ej}}=2.5$” means $V_{\mathrm{ej}}=2.5\;\sigma /t_{u}$.

## 4. Results

### 4.1. Ejection Velocity

**Figure 4:**
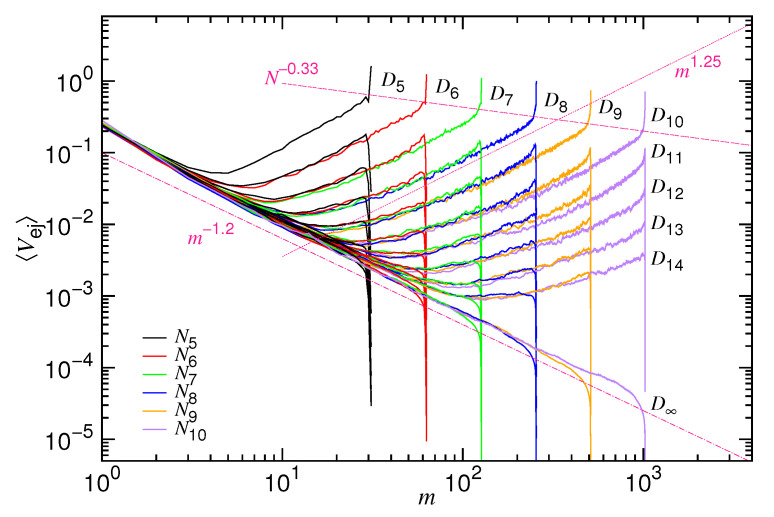
Averaged ejection velocity $\langle V_{\mathrm{ej}}\rangle$ vs. the number of the monomers *m* in the cavity. The chain length *N* is varied from $N_{5}=2^{5}$ to $N_{10}=2^{10}$, indicated in the legend. The cavity diameter *D* is varied from $D_{5}=\sqrt[{3}]{2.5\times 2^{5}}$ to $D_{14}=\sqrt[{3}]{2.5\times 2^{14}}$, indicated on the outer side of the branch curves. The cases with infinite diameter $D_{\infty }$ are plotted as references. The three scaling lines, $m^{1.25}$, $m^{-1.2}$, and $N^{0.33}$, are drawn in dark-pinked color.

We first investigate the mean ejection velocity $\langle V_{\mathrm{ej}}\rangle$ in an ejection process. The velocity is a function of the number of monomers *m* in the cavity. It can be calculated from the waiting time function. The waiting time function W(m) describes the dwelling time for an ejected chain to stay at a given state *m*. Thus, the average velocity at *m* can be calculated by taking the reciprocal of the waiting time function as σ/〈W(m)〉. Figure 4 presents the calculated velocity with *N* varied from $N_{5}=32$ to $N_{10}=1024$ and *D* varied from $D_{5}=2\sqrt[{3}]{10}$ to $D_{14}=16\sqrt[{3}]{10}$. The cases with infinite diameter $D_{\infty }$ are also studied; they are plotted in the figure as references.

We can see that $\langle V_{\mathrm{ej}}\rangle$ decreases first and then increases in a typical ejection process. Recall that, to follow the time evolution, the $\langle V_{\mathrm{ej}}\rangle$ curve should be traced from the right to the left of the figure. The two variational behaviors divide the ejection process into the mentioned confined stage and non-confined stage. At the confined stage, the velocity decreases, because the driving (owing to the confinement) reduces with time, due to the decrease of the number of monomers in the cavity. A scaling behavior of $m^{1.25}$ is observed. At the non-confined stage, $\langle V_{\mathrm{ej}}\rangle$ turns to show increasing behavior and scales as $m^{-1.2}$. It is because the rest of the chain occupies a space smaller than the cavity. There is no mechanical force to drive the chain anymore. In this situation, the system is driven by the thermodynamic (entropy) force from the external chain segments. The velocity increases, because the number of the external segments increases with time.

At a given chain length, increasing *D* reduces $\phi_{0}$ and, thus, decreases the confinement. Consequently, a decrease in the ejection velocity is expected. We do observe that the velocity decreases at the confined stage, with the whole curve moving downward in a parallel manner. However, at the non-confined stage, the velocity curve is basically not altered by increasing *D*. It shows that the mechanical influence does not last to the non-confined stage. All of the velocity curves evolve eventually toward the single curve profile for $D=D_{\infty }$.

If *D* is fixed, then we found that the $\langle V_{\mathrm{ej}}\rangle$ curves for different *N* join together to form a branch of curves at the confined stage. The branches $D_{5}$, $D_{6}$, ⋯, and $D_{14}$ can be seen in the figure. Our simulations show that the velocity is, in fact, slightly smaller for a longer chain in a given branch. It suggests a weak scaling dependence, $N^{x_{1}}$, on the chain length, as formulated in the denominator of Equation (8). The exponent $x_{1}$ is estimated to be 1/3 by varying the chain length at a fixed $\phi_{0}$. The departure velocity of ejection for $\phi_{0}=0.4$ exhibits a $N^{-0.33}$ behavior when *N* is varied from $N_{5}$ to $N_{10}$, as shown in the figure. The whole $\langle V_{\mathrm{ej}}\rangle$ curves look quite similar to the ones that are predicted in Figure 2. It asserts that Equation (8) can describe the scaling characteristics of ejection velocity well.

A physical explanation for $x_{1}=1/3$ is given below. When entering the pore, a monomer is subject to a drag from the chain segments in the cavity because of the chain connectivity. The drag effectively raises the friction coefficient by an amount of $\eta_{0}m^{x_{1}}$. Because the monomer is transferred from the three-dimensional cavity space into the one-dimensional pore channel, the exponent is expected to be 1/3, which reflects the change of the geometrical dimension [53]. In addition to the drag from the inside, there exists an impedance from the outside: The ejected monomers are accumulated near the pore exit, as a result of the fast ejection at the confined stage, and they have not yet been relaxed. It hinders the pore monomers to go outside and, therefore, the friction coefficient is raised by a second amount $\eta_{0}{\left(N-m\right)}^{x_{1}}$. Figure 4 has revealed that, at a given *m*, a longer chain on a $D_{i}$-branch exhibits a smaller velocity. It is clearly related to the impedance that is given by the accumulated monomers. Thus, the friction coefficient η for the pore monomers is about $\eta_{0}\left(m^{x_{1}}+{\left(N-m\right)}^{x_{1}}\right)$, which scales roughly as $\eta_{0}N^{x_{1}}$ if *N* is large.

The scaling exponents for $\langle V_{\mathrm{ej}}\rangle$ is $z_{1}=1/\left(3\nu -1\right)$ at the confined stage and $z_{2}=1+y_{2}$ at the non-confined stage. Here, we argue that $y_{2}$ is 2ν−1. At the confined stage, the monomers fill up the cavity space and have no net drift velocity. Thus, the dissipation of the free energy mainly occurs at the pore, dominated by the ejection velocity. As the process goes into the non-confined stage, the rest of the monomers can no longer fill up the cavity and exhibit a net drift velocity toward the pore. Thus, the dissipation is contributed from the *m* monomers inside the cavity and it reads as $m\cdot \eta_{0}V_{d}^{2}$. The drift velocity $V_{d}$ can be estimated by $dR_{m}/dt$, where $R_{m}∼\sigma m^{\nu }$ is the internal chain size, and, thus, is related to the ejection velocity by $m^{\nu -1}V_{\mathrm{ej}}$. The rate of dissipation is simply expressed by $\eta V_{\mathrm{ej}}^{2}$ in deriving Equation (5). To take the change into account, the friction coefficient should possess a scaling $\eta ∼\eta_{0}m^{2\nu -1}$, which gives $y_{2}=2\nu -1$. We have performed non-linear fitting for the velocity at N=256, 512 and 1024 for $D=D_{\infty }$ over a range of *m* between 10 and 0.8N. The obtained result is $m^{-1.21(3)}$, which is in good agreement with the prediction if one sets ν=0.6.

**Figure 5:**
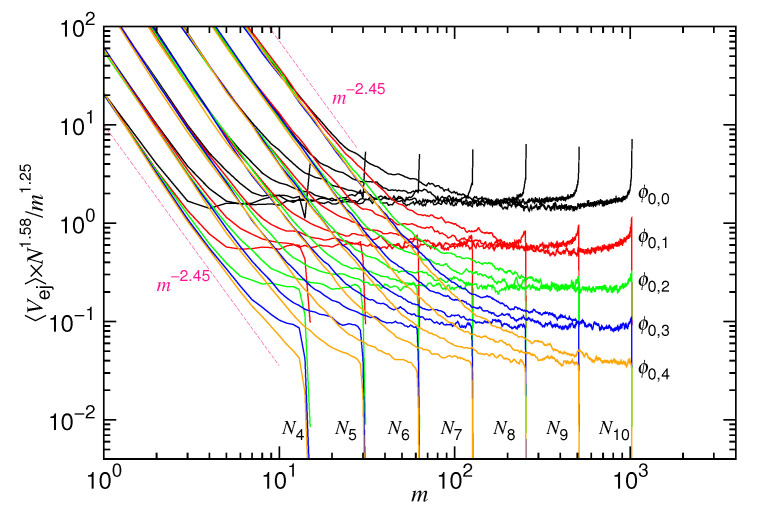
$\langle V_{\mathrm{ej}}\rangle \times N^{1.58}/m^{1.25}$ vs. *m* in an ejection process. The cases with the same initial volume fraction $\phi_{0}=\phi_{0,g}$ are plotted in the same color where $\phi_{0,g}=0.4\times 2^{-g}$. The chain length $N_{i}=2^{i}$ can be read near the bottom of the figure and the corresponding curves can be traced from there.

In order to make the scaling properties evident, we replot the $\langle V_{\mathrm{ej}}\rangle$ curves by multiplying the velocity with a factor $N^{x_{1}+z_{1}}/m^{z_{1}}$. Figure 5 presents the results, where $z_{1}=1.25$ and $x_{1}=0.33$ are used.

In the replot, the curves with the same generation number of $\phi_{0}$ are expected to show a horizontal branch at the confined stage. This is exactly what we observe in the figure. At the non-confined stage, the rescaled velocities, coming from a given *N* but different $\phi_{0}$, collapse together. The scaling exponent is found essentially $-(z_{1}+z_{2})=-2.45$. The results again ascertain that the velocity in an ejection process can be generally described by Equation (8).

**Figure 6:**
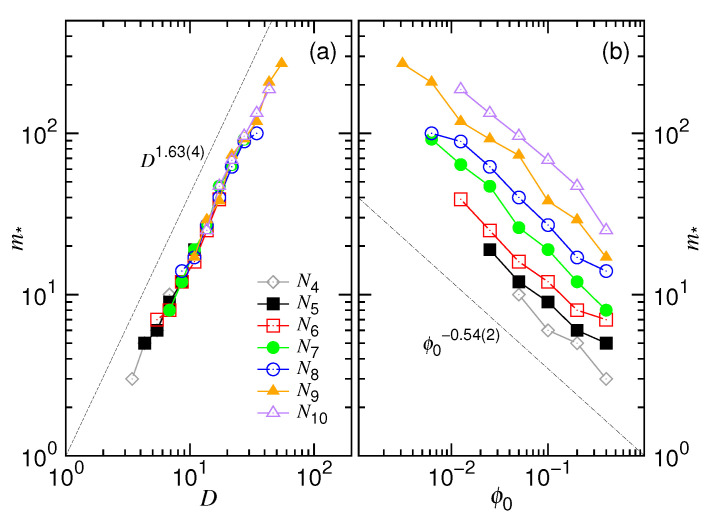
Critical monomer number $m_{*}$, which separates the confined and the non-confined stage in an ejection process, as a function of (**a**) the cavity diameter *D* and (**b**) the initial volume fraction $\phi_{0}$. The chain length can be read in the legend of (**a**) where $N_{i}=2^{i}$.

The critical monomer number $m_{*}$, which separates the confined and non-confined stage, can be determined by searching for the minimum of the $\langle V_{\mathrm{ej}}\rangle$ curve in Figure 4. The scaling behaviors are then studied by varying *D* and $\phi_{0}$, with *N* being fixed, as shown in Figure 6.

We can see that the $m_{*}$ data fall on a universal line in Panel (a) for different *N* values, with a fitting exponent equal to 1.63(4). It agrees with the theoretical description $D∼\sigma m_{*}^{\nu }$ or, equivalently, $m_{*}∼{\left(D/\sigma \right)}^{1/\nu }$. If the varying variable is $\phi_{0}$ (see Panel (b)), $m_{*}$ is found to show decreasing behavior with an exponent −0.54(2). The longer the chain length, the larger the $m_{*}$ value, and the $m_{*}$ curves are parallel for different *N*. The scaling behavior fulfills the description of $m_{*}∼{\left(N/\phi_{0}\right)}^{1/(3\nu )}$. Further verification for the scaling of $m_{*}$ against *N* can be found in the Supporting Information in Figure S1. It shows that $m_{*}∼N^{0.58(2)}$ at a fixed $\phi_{0}$. If *D* is fixed, then $m_{*}$ is essentially constant, scaling like $N^{0}$.

### 4.2. Time Variation of the Number of Monomers in the Cavity

**Figure 7:**
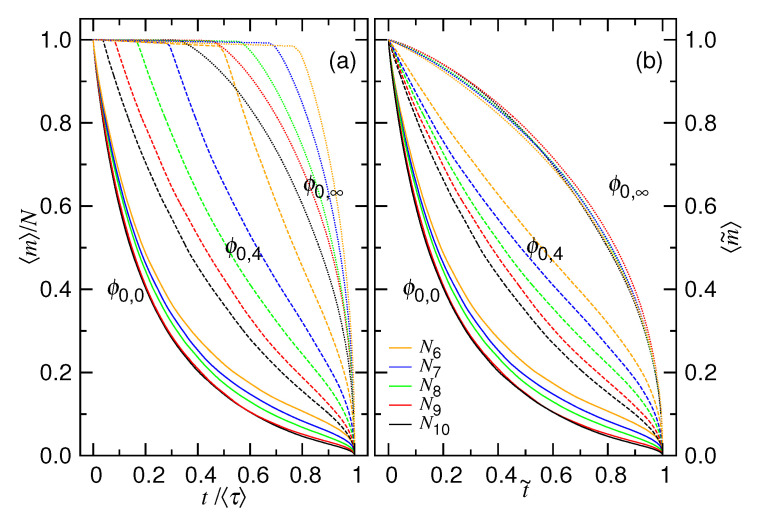
(**a**) 〈m〉/N versus t/〈τ〉 at $\phi_{0}=\phi_{0,0}$ (in solid line), $\phi_{0,4}$ (in dashed line), and $\phi_{0,\infty }$ (in dotted line) for different chain lengths. Different colors denote different chain lengths, which can be read in the legend of Panel (**b**) with $N_{i}=2^{i}$. The three $\phi_{0}$ values are $\phi_{0,0}=0.4$, $\phi_{0,4}=0.025$, and $\phi_{0,\infty }=0.0$. (**b**) Replot of the curves in Panel (**a**) by trimming the the plateau region. The definitions of the two coordinate variables are $\tilde{t}=\left(t-\langle \tau_{n}\rangle \right)/\left(\langle \tau \rangle -\langle \tau_{n}\rangle \right)$ and $\langle \tilde{m}\rangle =\left(\langle m\rangle -m_{n}\right)/\left(N-m_{n}\right)$.

The evolution of the averaged number of monomers 〈m〉 in the cavity with time is studied in this subsection. In order to compare the results across different simulation conditions, the monomer number is normalized by the chain length *N* and time is normalized by 〈τ〉. Here, 〈τ〉 is the mean time that is needed to transport all of the monomers from the cavity to the outside since the beginning. The results, 〈m〉/N vs. t/〈τ〉, for different $\phi_{0}$ values, are presented in Figure 7a.

At $\phi_{0}=0.4$ (that is $\phi_{0,0}$), the curve decreases immediately. A longer chain is found to give a faster decreasing behavior. As $\phi_{0}$ reduces, a plateau appears and extends on the curve, prior to the happening of the fast decreasing. We noticed that, following the plateau, the 〈m〉 value is only decreased by 1, which corresponds to the pore length $L_{p}=1.0$. It suggests that considerable time is spent for the head monomer to go across the pore and search for the exit. The appearance of the plateau can be interpreted as a formation of nucleus before entering to the phase of a true ejection, in analogue of a nucleation phenomenon [54,55]. After reaching the critical nucleus size, which, here, is 1, the number of monomers outside the cavity can grow continuously without stalling, resulting in a smooth decreasing 〈m〉/N curve, as shown in the figure. It is analogous to the growth of a crystal after nucleation. The smaller the initial volume fraction, the longer the nucleation time, upper bounded by the one at the zero initial volume fraction $\phi_{0,\infty }$. Here, the nucleation time is defined to be the mean time that is required for a system to produce the critical nucleus size, i.e., for one monomer to leave the cavity in this case.

In order to properly investigate the evolution of the number of the monomers in an ejection process, we trim the mean nucleation time $\langle \tau_{n}\rangle$. Figure 7b presents the new plots while using the trimmed and normalized variables: $\tilde{t}=\left(t-\langle \tau_{n}\rangle \right)/\langle \tau_{\mathrm{ej}}\rangle$ and $\langle \tilde{m}\rangle =\left(\langle m\rangle -m_{n}\right)/\left(N-m_{n}\right)$, where $\langle \tau_{\mathrm{ej}}\rangle =\langle \tau \rangle -\langle \tau_{n}\rangle$ and $m_{n}=L_{p}/\sigma$ is the critical nucleus size. We can see that the plateau is gone and the normalized 〈m〉 value decreases directly without stalling. At $\phi_{0}=\phi_{0,\infty }$, the decreasing curve is completely concave and not sensitive to the chain length. It fulfills the description of Equation (7), where $M_{0}$ is approximately *N* at the zero volume fraction. For the other two cases, $\phi_{0}=\phi_{0,0}$ and $\phi_{0}=\phi_{0,4}$, the main portion of the curve is convex and it can be described by Equation (3).

**Figure 8:**
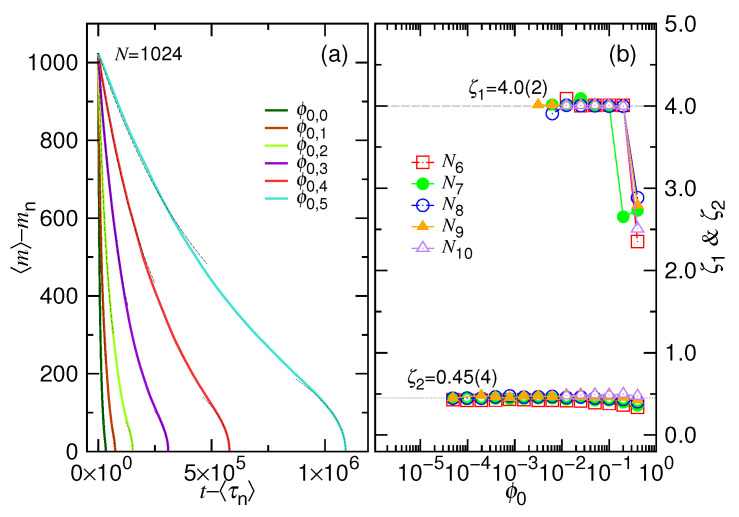
(**a**) Variation of the trimmed mean monomer number $\langle m\rangle -m_{n}$ against the time $t-\langle \tau_{n}\rangle$. The chain length is N=1024. The $\phi_{0}$ value is given in the legend with $\phi_{0,g}=0.4\times 2^{-g}$. The curves are fit by Equation (3) from the starting side, while fit by Equation (7) from the ending side. The fitting curves are plotted in dashed and dotted lines, respectively. (**b**) the obtained $\zeta_{1}$ and $\zeta_{2}$ exponents are plotted as function of $\phi_{0}$. The chain length $N_{i}=2^{i}$ is given in the legend.

Analysis is done by studying the trimmed “$\langle m\rangle -m_{n}$ vs. $t-\langle \tau_{n}\rangle$”curves. The starting portion of the curves is fit by Equation (3) with the two fitting parameters $\zeta_{1}$ and $t_{0}$, while the terminating portion is fit by Equation (7) with $\zeta_{2}$ and $M_{0}$ being the parameters. An example of fitting is given in Figure 8a, where the chain length is 1024. We can see that the trimmed 〈m〉 curves can be well fit by the two equations, as shown in dashed and dotted black lines, from the two sides of the curves.

Figure 8b plots the obtained $\zeta_{1}$ and $\zeta_{2}$ exponents as a function of $\phi_{0}$ at different chain lengths. We found that the two exponents converge to a value of 4.0(2) and 0.45(4), respectively. It agrees well with the theoretical predictions: $\zeta_{1}=1/\left(z_{1}-1\right)$ and $\zeta_{2}=1/\left(z_{2}+1\right)$, with $z_{1}=1.25$ and $z_{2}=1.2$.

**Figure 9:**
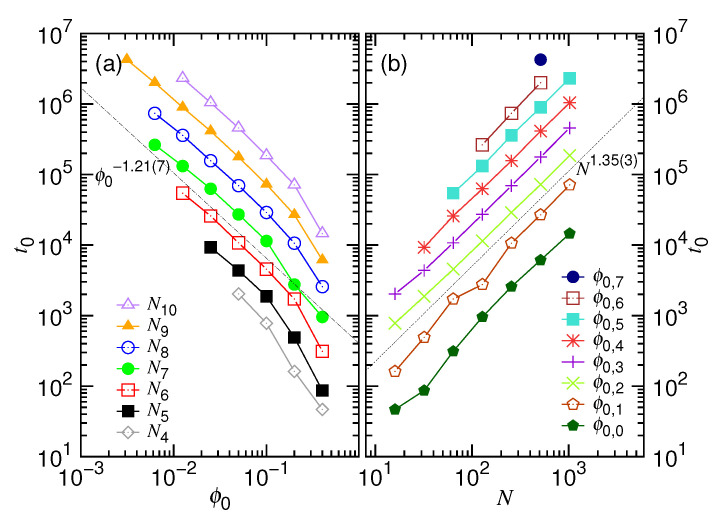
Fitting parameter $t_{0}$ for Equation (3) as a function of (**a**) the initial volume fraction $\phi_{0}$ and (**b**) the chain length *N*. The parameter *N* is fixed in Panel (**a**), while $\phi_{0}$ is fixed in Panel (**b**). The values of the parameters are given in the legends through the formula $N_{i}=2^{i}$ and $\phi_{0,g}=0.4\times 2^{-g}$.

Figure 9 presents the scaling behaviors of the $t_{0}$ parameter for Equation (3).

Panels (a) and (b) show that $t_{0}$ scales as $\phi_{0}^{-1.21(7)}N^{1.35(3)}$. Recall that our theory predicts $t_{0}∼\phi_{0}^{-z_{1}}N^{1+x_{1}}\Delta t_{0}$. Again, the results are consistent with the prediction by setting $z_{1}$ and $x_{1}$, respectively, to the values 1.25 and 1/3.

**Figure 10:**
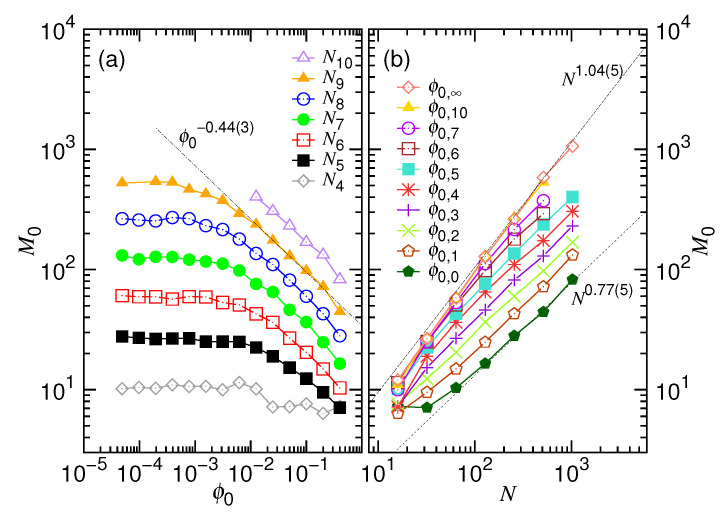
Fitting parameter $M_{0}$ for Equation (7) as a function of (**a**) the initial volume fraction $\phi_{0}$ and (**b**) the chain length *N*. The values of the fixed parameters *N* and $\phi_{0}$ are given in the legends of the two panels, respectively, through the formula $N_{i}=2^{i}$ and $\phi_{0,g}=0.4\times 2^{-g}$.

Figure 10 presents the variation of the obtained $M_{0}$ parameter for Equation (7). The parameter is expected to behave as ${\langle \tau_{\mathrm{ej}}\rangle }^{\zeta_{2}}$.

The scaling looks similar to the ones in Figure 13a,e, shown later in Section 4.3, with the exponents being multiplied by a factor $\zeta_{2}$. For example, the scaling exponent is −0.44(3) in Panel (a) when $M_{0}$ varies in the large $\phi_{0}$ region. It is close to the predicted value 0.5. The *N*-exponents changes from 0.77(5) to 1.04(5) as $\phi_{0}$ decreases. It agrees well with the expected values, 0.72 and 1.0, respectively. We will explain these behaviors later.

**Figure 11:**
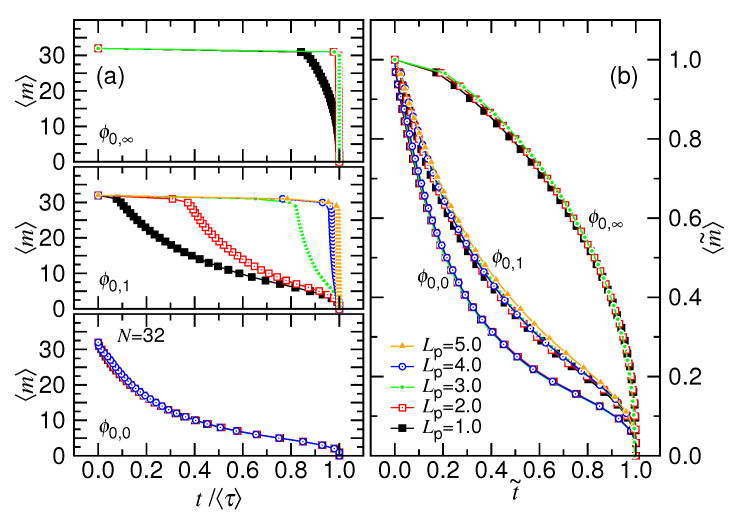
(**a**) 〈m〉 versus t/〈τ〉 for N=32 at the three $\phi_{0}$ values: 0.4 (in the bottom panel), 0.2 (in the middle panel), and 0.0 (in the top panel). The pore length $L_{p}$ is varied and the value is given in the legend of Panel (**b**). (**b**) Replot of the time variation of the number of monomers in the cavity using the re-scaled coordinate variables: $\tilde{t}=\left(t-\langle \tau_{n}\rangle \right)/\left(\langle \tau \rangle -\langle \tau_{n}\rangle \right)$ and $\langle \tilde{m}\rangle =\left(\langle m\rangle -m_{n}\right)/\left(N-m_{n}\right)$.

We performed simulations by varying the pore length in order to understand whether the stalling occurred prior to the ejection shows characteristics of nucleation. Figure 11a shows the variation of 〈m〉 against *t* at three $\phi_{0}$ values. The chain length is 32 and pore length $L_{p}$ is varied from 1.0 to 5.0.

Using the line-point plot, we can see that the plateau becomes wider with increasing $L_{p}$ at $\phi_{0}=\phi_{0,1}$, and the value of 〈m〉 is decreased by a small value, approximately $L_{p}/\sigma$. It shows that the critical nucleus size is directly related to the pore length. Similar behavior can be seen for the case with $\phi_{0}=\phi_{0,\infty }$. Shifting the time and the monomer number to be $t-\langle \tau_{n}\rangle$ and $\langle m\rangle -m_{n}$, respectively, we eliminate the plateau from the curve. The curves are then replotted in Figure 11b using the normalized coordinates $\tilde{t}$ and $\langle \tilde{m}\rangle$. We discover that the curves collapse together for different pore lengths at the same $\phi_{0}$ value and follow the description of the ejection equations. It demonstrates that the plateau is a stage independent of the following ejection process.

We further find that the plateau region does not show up at $\phi_{0}=0.4$, as seen in the bottom panel of Figure 11a. In this case, no nucleation is required before the ejection and, therefore, $m_{n}=0$ and $\langle \tau_{n}\rangle =0.0$. The reason for the absence of the nucleation can be understood, as follows. The osmotic pressure for a monomer to be presented in the pore channel is estimated to be $\Pi_{p}=k_{B}T/\left(\pi r_{p}^{2}\sigma \right)$, which has a value of $0.566\;k_{B}T/\sigma^{3}$ by plugging in the pore radius $r_{p}=0.75\;\sigma$ of this study. The osmotic pressure of monomers in the cavity, on the other hand, can be calculated by $\Pi_{c}=k_{B}T\phi_{0}/\left(\frac{1}{6}\pi \sigma^{3}\right)$. At $\phi_{0}=0.4$, the interior osmotic pressure $\Pi_{c}$ is $0.764\;k_{B}T/\sigma^{3}$, which is higher than $\Pi_{p}$. Therefore, the ejection can proceed in an imminent way since the starting of the process. For the other studied cases $\phi_{0}=\phi_{0,g}$ with g≥1, $\Pi_{c}$ is smaller than $\Pi_{p}$. The heading monomers need to overcome the energy barrier that is created by the osmotic pressure difference to go outside. Consequently, a nucleation-like phenomenon appears, as we have observed in Figure 7 and Figure 11. When the monomer enters the outer semi-space, the osmotic pressure drops to zero and the process turns to follow the ejection description that is given in Section 2. We have verified that the nucleation stage appears at a lower $\phi_{0}$ value if the pore radius $r_{p}$ is increased. It firmly supports that the nucleation is determined by the osmotic pressure difference.

**Figure 12:**
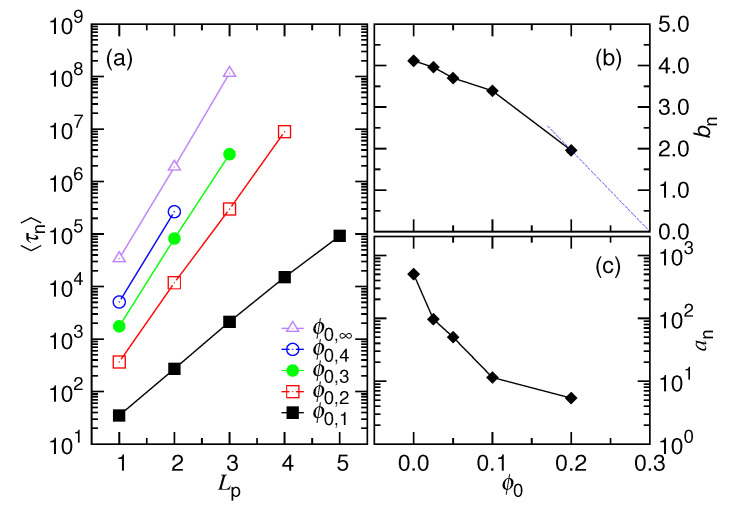
(**a**) Average nucleation time $\langle \tau_{n}\rangle$ versus the pore length $L_{p}$ at different $\phi_{0}$. The $\phi_{0}$ value can be calculated by the formula $\phi_{0,g}=0.4\times 2^{-g}$, read in the legend. We fit the $\langle \tau_{n}\rangle$ curve by the equation $a_{n}\exp\left(b_{n}L_{p}\right)$. The results of the fitting, $b_{n}$ and $a_{n}$, are plotted in Panels (**b**) and (**c**), respectively, as a function of $\phi_{0}$.

Figure 12a plots the variation of the nucleation time $\langle \tau_{n}\rangle$ against the pore length $L_{p}$. We find that $\langle \tau_{n}\rangle$ grows exponentially with $L_{p}$, because the data are linear when plotted with a logarithmic scale on the *y*-axis.

It suggests a Kramers’ escape problem, which gives the nucleation time $\langle \tau_{n}\rangle ∼\frac{\sigma^{2}\eta }{k_{B}T}\exp\left(\frac{L_{p}\Delta \mu_{\mathrm{cp}}}{\sigma k_{B}T}\right)$, where $\Delta \mu_{\mathrm{cp}}=\mu_{p}-\mu_{c}$ is the chemical potential difference between the cavity and pore. When an ejection begins, the heading $L_{p}/\sigma$ monomers on the chain have to traverse the pore channel first. A total amount of energy $\left(L_{p}/\sigma \right)\Delta \mu_{\mathrm{cp}}$ is required for boosting the system. This energy is the activation energy $E_{a}$ and the rate of escape over it is the key to understanding the problem. Kramers has predicted the escape rate to be $\rho ∼\eta^{-1}\exp\left(-E_{a}/k_{B}T\right)$ under a condition of large viscosity [56,57]. The nucleation time formula that is given here is obtained by taking the reciprocal of the escape rate. In this study, $\langle \tau_{n}\rangle$ is called “the nucleation time”, rather than “the escape time”. We do it to emphasize the similarity of the phenomena with the nucleation. The Kramers’ escape theory that is used here is for estimating the formation time of a “nucleus”.

The nucleation time $\langle \tau_{n}\rangle$ is studied by fitting with the function $a_{n}\exp\left(b_{n}L_{p}\right)$. Figure 12b,c provides the fitting parameters $b_{n}$ and $a_{n}$. Both $b_{n}$ and $a_{n}$ decrease with increasing $\phi_{0}$. Noticeably, the extrapolation of the $b_{n}$ curve shows a tendency to hit zero at a $\phi_{0}$ value around 0.3. It defines the threshold for the disappearance of a nucleation. The value corresponds well to the local volume fraction of a monomer in the pore channel, which is $\phi_{p}=\left(\frac{\pi }{6}\sigma^{3}\right)/\left(\pi r_{p}^{2}\sigma \right)=0.296$ in this study. The results suggest that $\Delta \mu_{\mathrm{cp}}$ is a function of the osmotic pressure difference $\Delta \Pi_{\mathrm{cp}}=\Pi_{p}-\Pi_{c}$. Figure 12b further reveals that $b_{n}$ is approximately linear with $\Delta \phi_{0}=\phi_{0p}-\phi_{0c}$, with the drawing of the blue dashed line. Because the osmotic pressure is proportional to the volume fraction to the first order, we conjecture that the chemical potential difference is approximately linear with $\Delta \Pi_{\mathrm{cp}}$.

### 4.3. Ejection Time and Nucleation Time

**Figure 13:**
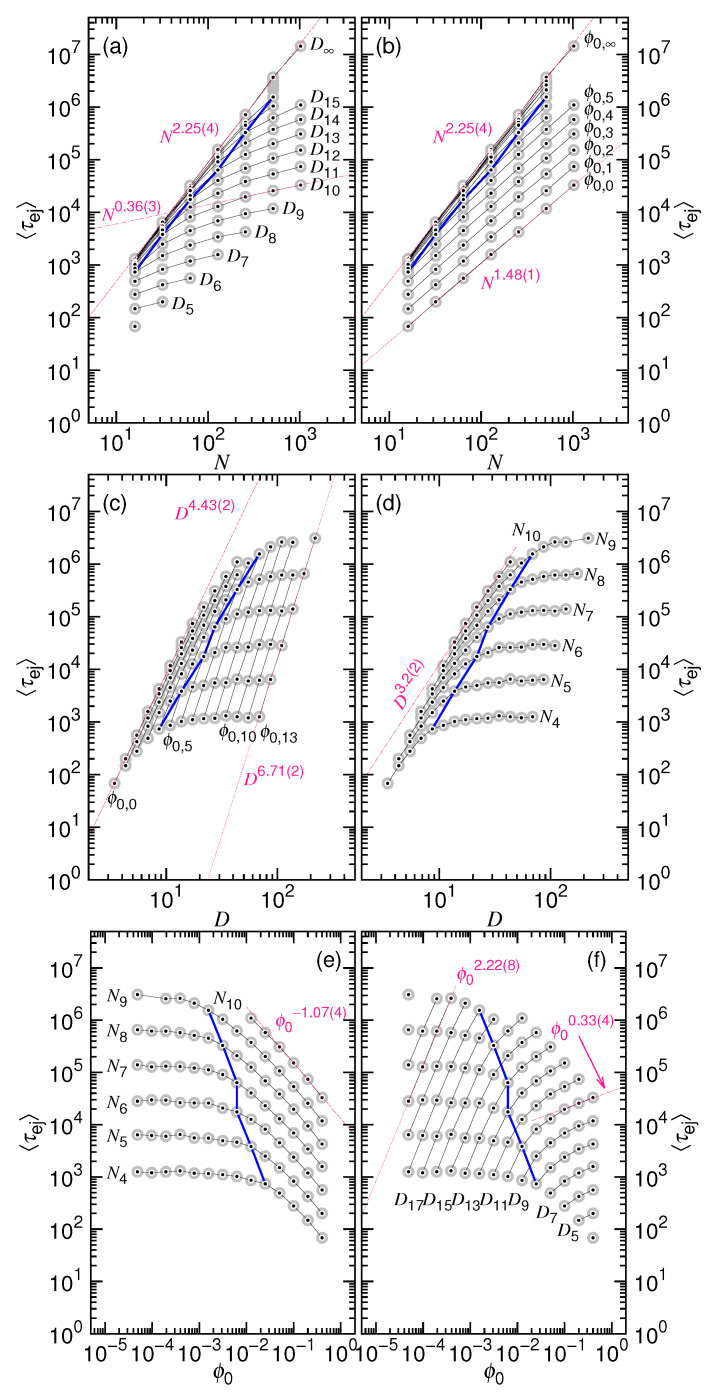
(**a**) Average ejection time $\langle \tau_{\mathrm{ej}}\rangle$ vs. chain length *N* at fixed cavity diameter *D*; (**b**) $\langle \tau_{\mathrm{ej}}\rangle$ vs. *N* at fixed initial volume fraction $\phi_{0}$; (**c**) $\langle \tau_{\mathrm{ej}}\rangle$ vs. *D* at fixed $\phi_{0}$; (**d**) $\langle \tau_{\mathrm{ej}}\rangle$ vs. *D* at fixed *N*; (**e**) $\langle \tau_{\mathrm{ej}}\rangle$ vs. $\phi_{0}$ at fixed *N*; (**f**) $\langle \tau_{\mathrm{ej}}\rangle$ vs. $\phi_{0}$ at fixed *D*. The data are connected by fixing a parameter, indicated near the curve. The value of the parameter can be calculated by the formulas: $N_{i}=2^{i}$, $D_{j}=\sqrt[{3}]{2.5\times 2^{j}}$, and $\phi_{0,g}=0.4\times 2^{-g}$. The blue line indicates the location of the ejection time occurred at the critical point. Dark-pink lines show noticed scaling behaviors.

Following the analysis of the previous subsection, we decompose the processing time 〈τ〉 into the two parts, the nucleation time $\langle \tau_{n}\rangle$ and the ejection time $\langle \tau_{\mathrm{ej}}\rangle$, and separately study their scaling behaviors. Figure 13 presents the variations of $\langle \tau_{\mathrm{ej}}\rangle$ as a function of the chain length *N* (in Panels (a) and (b)), the cavity diameter *D* (in Panels (c) and (d)), and the initial volume fraction $\phi_{0}$ (in Panels (e) and (f)). We recall that the three variables, *N*, *D*, and $\phi_{0}$, are not completely independent, because $\phi_{0}=N{\left(\sigma /D\right)}^{3}$. Thus, the ejection time can be expressed in terms of any pair of the three variables.

Two distinguishable scaling behaviors are observed in Figure 13a. When *D* is small, $\langle \tau_{\mathrm{ej}}\rangle$ shows slow growing behavior with *N* as $N^{0.36(3)}$. An increasing *D* moves upward the $\langle \tau_{\mathrm{ej}}\rangle$ curve. The paralleled curves are observed to deflect downward, from the small *N* side, to show a steepened variation; the scaling changes to $N^{2.25(4)}$. The picture is given here: decreasing *N* eventually sends the chain into the non-confined stage at a given *D* value. In that situation, the ejection is similar to a pure translocation and, thus, follows the strong increasing behavior, like the $D_{\infty }$ case.

If the $\phi_{0}$ value is large and fixed (refer to Figure 13b), then the scaling of $\langle \tau_{\mathrm{ej}}\rangle$ is $N^{1.48(1)}$. The curve also moves upward in a parallel way with decreasing $\phi_{0}$, and it deflects to follow $N^{2.25(4)}$ when approaching the limiting line that is defined by $\phi_{0,\infty }$. The data that are connected by the blue segments in the plots indicate the critical chain length $N_{*}$, which was obtained by studying the velocity curves in Figure 4. When $N<N_{*}$, the ejection velocity increases monotonically in a process and it has no minimum. Consequently, above the blue connected data, the process is only proceeded via the non-confined stage, while, below the data, it passes the two stages with the confined stage being the dominated one.

The results that are presented here look quite similar to the theoretical curves given in Figure 1, where the exponent of *N* is predicted to change from $x_{1}$ to $1+z_{2}$ with increasing *D* in Panel (a) and from $\frac{2}{3\nu }+x_{1}$ to $1+z_{2}$ with decreasing $\phi_{0}$ in Panel (b). The exponents that are extracted from the simulations are $x_{1}=0.36\left(3\right)$ and $z_{2}=1.25\left(4\right)$. They are very close to the theoretical values $x_{1}=1/3$ and $z_{2}=2\nu$.

Figure 13c provides the variation of $\langle \tau_{\mathrm{ej}}\rangle$ with *D* under the $\phi_{0}$-fixed condition. The time curve scales as $D^{4.43(2)}$ at $\phi_{0}=0.4$, and then moves to the right with decreasing $\phi_{0}$. The behavior eventually changes to $D^{6.71(2)}$ as $\phi_{0}$ becomes small. The demarcation is indicated by the blue connected line on the plot that shows the location of the critical diameter $D_{*}$. On the right side of the line, the chain feels free in the cavity; on the left side, the chain suffers the confinement of the cavity and the pressing strongly influences the ejection. The observed scaling behaviors are in agreement with Figure 1c. The *D*-exponent is expected to be $\frac{2}{\nu }+3x_{1}$ at large $\phi_{0}$ and it turns to be $3(1+z_{2})$ as the confinement disappears.

If it is the chain length *N* being fixed (refer to Figure 13d), we can see that $\langle \tau_{\mathrm{ej}}\rangle$ increases with *D* with a scaling exponent that is equal to 3.2(2) in the confinement region. It agrees with the prediction $D^{2/\nu }$ of Figure 1d. When entering to the non-confined region (the region right to the blue line), the time curves are quickly leveled off. It shows that the ejection of chain is no more affected by the cavity wall.

Figure 13e shows how $\langle \tau_{\mathrm{ej}}\rangle$ varies with $\phi_{0}$ for a given *N*. It is essentially a replot of Figure 13d by reversing the direction of the *x*-axis in the log-log plot via the following coordinate mapping: $D⟶\phi_{0}=N\sigma^{3}D^{-3}$. On the left side of the blue demarcation line, the ejection time is constant and not affected by the cavity size. On the right side, $\langle \tau_{\mathrm{ej}}\rangle$ decreases with increasing $\phi_{0}$ and the obtained exponent is −1.07(4), being consistent with the theoretical value $-\frac{2}{3\nu }$.

With *D* being fixed, the plot of Figure 13f reveals different scaling behaviors. In the region $\phi_{0}<\phi_{*}$ (left to the blue line), a power-law growth $\phi_{0}^{2.22(8)}$ is found for $\langle \tau_{\mathrm{ej}}\rangle$. In the region $\phi_{0}>\phi_{*}$ (right to the blue line), the growth slows down and a smaller exponent 0.33(4) is observed. Our scaling theory states a consistent result, with the exponent being $1+z_{2}$ and $x_{1}$, respectively (refer to Figure 1f).

**Figure 14:**
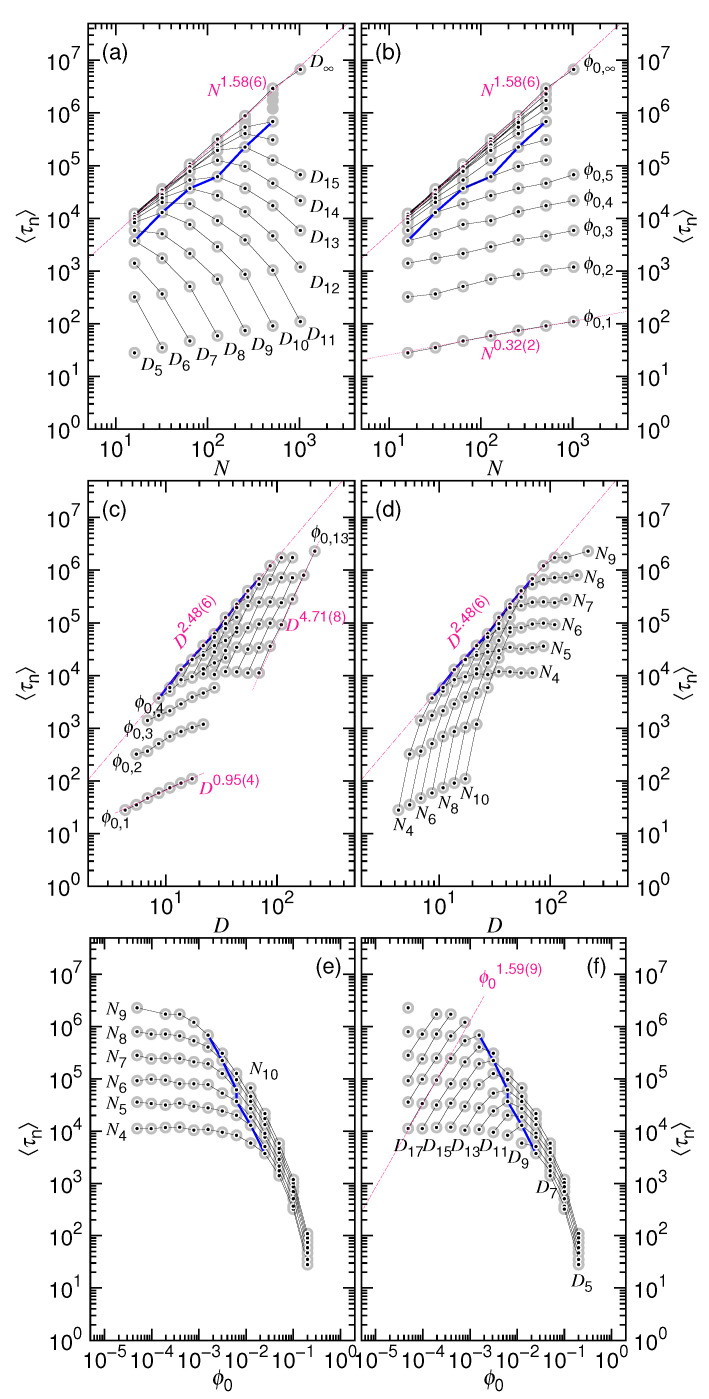
(**a**) Average nucleation time $\langle \tau_{n}\rangle$ vs. *N* at fixed *D*; (**b**) $\langle \tau_{n}\rangle$ vs. *N* at fixed $\phi_{0}$; (**c**) $\langle \tau_{n}\rangle$ vs. *D* at fixed $\phi_{0}$; (**d**) $\langle \tau_{n}\rangle$ vs. *D* at fixed *N*; (**e**) $\langle \tau_{n}\rangle$ vs. $\phi_{0}$ at fixed *N*; (**f**) $\langle \tau_{n}\rangle$ vs. $\phi_{0}$ at fixed *D*. The data are connected by fixing a variable, indicated near the curve. The value of the variable can be calculated by the formulas: $N_{i}=2^{i}$, $D_{j}=\sqrt[{3}]{2.5\times 2^{j}}$, and $\phi_{0,g}=0.4\times 2^{-g}$. The blue line indicates the location of the nucleation time occurred at the critical point. Dark-pinked lines show noticed scaling behaviors.

Figure 14 presents how the average nucleation time $\langle \tau_{n}\rangle$ varies with *N*, *D*, and $\phi_{0}$.

Panel (a) shows that $\langle \tau_{n}\rangle$ decreases with increasing *N* in the region $N>N_{*}$ (below the blue demarcation line) if *D* is fixed. It follows the intuition that the osmotic pressure in the cavity increases with the chain length and, therefore, the energy barrier to overcome inside the pore becomes smaller, which reduces the nucleation time. In the region above the blue line ($N<N_{*}$), the chain size is smaller than the cavity size, so the chain is not suffered from the pressing of the cavity. A longer chain gives a stronger drag to the head monomer, which increases the resistance for the chain in order to traverse the pore. Therefore, the nucleation time increases. Our simulation shows that $\langle \tau_{n}\rangle ∼N^{1.58(6)}$ at the infinite *D*.

A physical explanation is given below. The nucleation time is described by the Kramers equation $\langle \tau_{n}\rangle ≃\frac{\sigma^{2}\eta }{k_{B}T}\exp\left(\frac{L_{p}\Delta \mu_{\mathrm{cp}}}{\sigma k_{B}T}\right)$. For the cases with $D_{\infty }$, the chemical potential difference $\Delta \mu_{\mathrm{cp}}$ is a constant and it does not change with the chain length. Thus, the variation of $\langle \tau_{n}\rangle$ is contributed from the change of η, which is the effective friction coefficient for the head monomer to traverse the pore. The value of η is directly proportional to the drag exerting on the head monomer, which is $\eta_{0}N$, multiplying a geometrical restriction factor for the chain coil to go into the one-dimensional pore, being estimated to be $N^{1/d_{f}}$, where $d_{f}=1/\nu$ is the fractal dimension of the chain coil [53]. Consequentlys, η scales with *N* with a scaling exponent equal to 1+ν, which is in good agreement with our observation.

If $\phi_{0}$ is fixed in the study, as shown in Figure 14b, the scaling is found to follow $N^{0.32(2)}$, as $\phi_{0}$ is large, for example, at $\phi_{0}=0.2$. A similar physical picture can be used in order to explain the behavior. Because $\Delta \mu_{\mathrm{cp}}$ is constant under the $\phi_{0}$-fixed condition, the dependence of $\langle \tau_{n}\rangle$ on *N* merely comes from the variation of η. In this situation, the drag of the chain body on the head monomer is “blocked” by the confinement; η is only contributed from the geometrical restriction factor, which is $N^{1/3}$, because the fractal dimension of the confined chain is three. As $\phi_{0}$ decreases, we find that the nucleation time curve moves upward in a parallel manner. The left portion of the curve deflects when intercepting with the $\phi_{*}$ line and it turns to follow the scaling for the null $\phi_{0}$ case.

Figure 14c is related to Figure 14b by mapping the abscissa from *N* to *D* via the relation $N=\phi_{0}{\left(D/\sigma \right)}^{3}$. Therefore, the scaling behaviors are expected to be $D^{1}$ at large $\phi_{0}$ and $D^{3(1+\nu )}$ at small $\phi_{0}$. We obtained a consistent simulation result with the exponent being 0.95(4) and 4.71(8) for the two situations, respectively. It is worth noticing that, with decreasing $\phi_{0}$ from the large value, the time curve moves upward and deflects as it touches the blue demarcation line at $D=D_{*}$. It looks like the up-moving curve is reflected by the demarcation line and bounced to the right-hand side. We have measured the demarcation line, which exhibits a scaling of $D^{2.48(6)}$.

If *N* is fixed (refer to Figure 14d), we can see that $\langle \tau_{n}\rangle$ drastically increaseswith *D* and is leveled off after touching the demarcation line. The leveling-off value defines the upper bound of the nucleation time for a given *N*, which occurs when the cavity is too large to impose a real confinement pressure in order to help the ejection.

Figure 14e is basically an abscissa-reversed plot of Figure 14d. A notable difference is that the drastically diminished curves in the large $\phi_{0}$ region tend to bundle together and become nulled (which is negative infinity in the log-log plot) at a $\phi_{0}$ value of around 0.3. It corresponds well to the picture that is described in the previous subsection that the nucleation occurs when the initial volume fraction in the cavity, $\phi_{0}$, is smaller than the volume fraction of a monomer that is presented in the pore, which is $\phi_{p}≃0.296$ in this study. The nucleation time drops to zero at a different place in Figure 14d, which is estimated at $D=\sigma {\left(N/\phi_{p}\right)}^{1/3}$. Therefore, the dropping curves appear in parallel to each other for the different chain lengths.

The last panel of the figure, Panel (f), shows the variation of the nucleation time with respect to $\phi_{0}$ at different cavity sizes. It can be related to Panel (a) by mapping *N* to $\phi_{0}$ via the equation $N=\phi_{0}{\left(D/\sigma \right)}^{3}$. The observed scaling $\phi_{0}^{1.59(9)}$ in the small $\phi_{0}$ region corresponds to the behavior $N^{1+\nu }$ in Panel (a), because *N* scales directly with $\phi_{0}$ if *D* is fixed. The results also reveal that the nucleation time first increases with $\phi_{0}$ and then decreases. The peak of the transition is located at $\phi_{0}=\phi_{*}$.

## 5. Discussions and Conclusions

**Figure 15:**
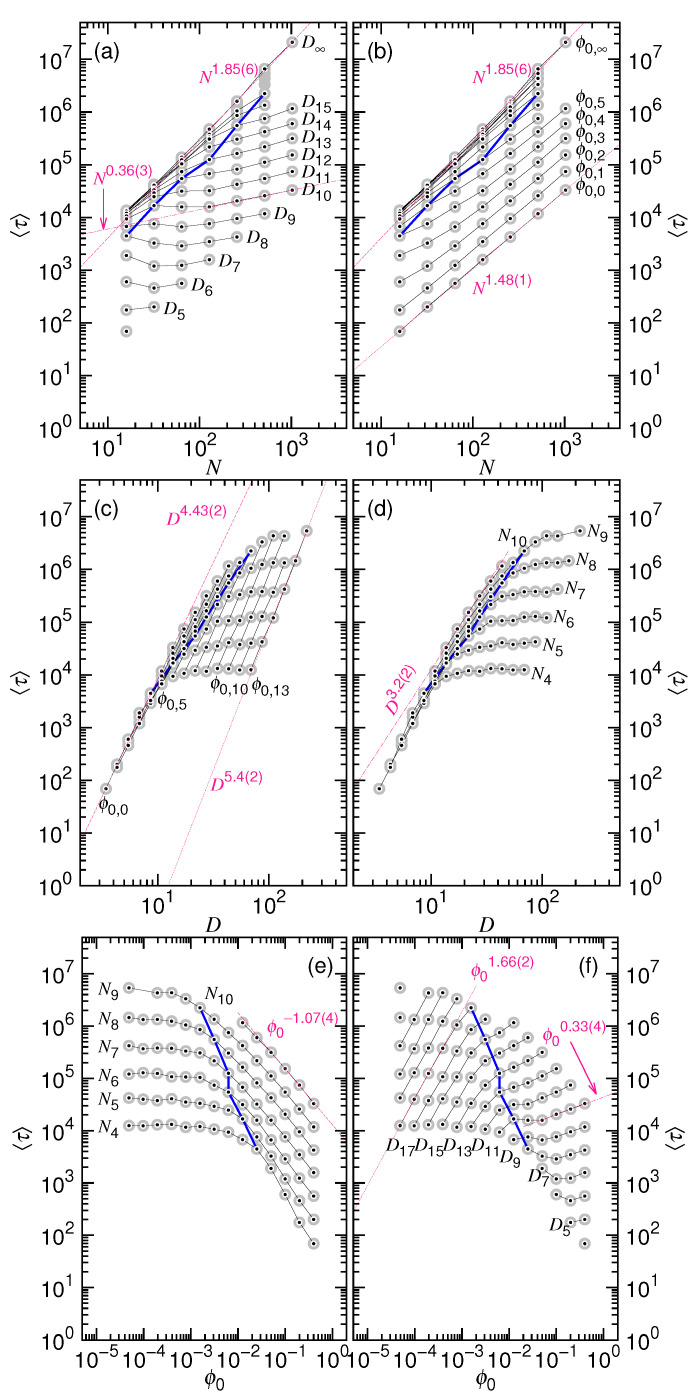
(**a**) Average total time $\langle \tau \rangle =\langle \tau_{\mathrm{ej}}\rangle +\langle \tau_{n}\rangle$ vs. *N* at fixed *D*; (**b**) 〈τ〉 vs. *N* at fixed $\phi_{0}$; (**c**) 〈τ〉 vs. *D* at fixed $\phi_{0}$; (**d**) 〈τ〉 vs. *D* at fixed *N*; (**e**) 〈τ〉 vs. $\phi_{0}$ at fixed *N*; (**f**) 〈τ〉 vs. $\phi_{0}$ at fixed *D*. The data are connected by fixing a variable, indicated near the curve. The value of the variable can be calculated by the formulas: $N_{i}=2^{i}$, $D_{j}=\sqrt[{3}]{2.5\times 2^{j}}$, and $\phi_{0,g}=0.4\times 2^{-g}$. The blue line indicates the location of the total time occurred at the critical point. Dark-pinked lines show noticed scaling behaviors.

Our simulations have showed that the order of the nucleation time $\langle \tau_{n}\rangle$ can be as large as the ejection time $\langle \tau_{\mathrm{ej}}\rangle$. Thus, it is important to separate the two time and study their properties properly. An analysis using the total processing time $\langle \tau \rangle =\langle \tau_{n}\rangle +\langle \tau_{\mathrm{ej}}\rangle$ in order to study the scaling behavior of ejection should be not accurate. It might explain why the scaling exponents that are reported in literature are not always consistent [27,28], because people usually thought that the nucleation time is negligible and would not influence greatly the ejection or translocation time. In order to demonstrate the influences, we present in Figure 15 the total processing time 〈τ〉 as a function of *N*, *D*, and $\phi_{0}$.

We can see that the scaling behavior is significantly affected in the small $\phi_{0}$ or large *D* region due to of the growing importance of the nucleation. For example, 〈τ〉 gives a underestimated scaling $N^{1.85(6)}$ at $D=D_{\infty }$ in Figure 15a, as compared to $N^{2.25(4)}$ in Figure 13a, owing to the mixture with the weak scaling $N^{1.58(6)}$ of $\langle \tau_{n}\rangle$. The *D*-exponent for 〈τ〉 is 5.4(2) at $\phi_{0}=\phi_{0,13}$ in Figure 15c, being significantly smaller than 6.71(2) for $\langle \tau_{\mathrm{ej}}\rangle$ in Figure 13c. Rather than showing $\phi_{0}^{2.22(8)}$ behavior in Panel (f) at small $\phi_{0}$, the 〈τ〉 curve exhibits a weaker scaling $\phi_{0}^{1.66(2)}$, because of the influence of $\langle \tau_{n}\rangle$. Moreover, the transition between the two scalings, deflecting from one branch of the paralleled curves to the other, appears less neatly in Figure 15. For example, the time curves are not so in parallel and curve up in the small *N* region in Panels (a) and (b). The decreasing portions of the curves in Panel (e) look less paralleled in comparison with Figure 13e. The results show that the scaling cannot be accurately studied without removing the nucleation time.

**Figure 16:**
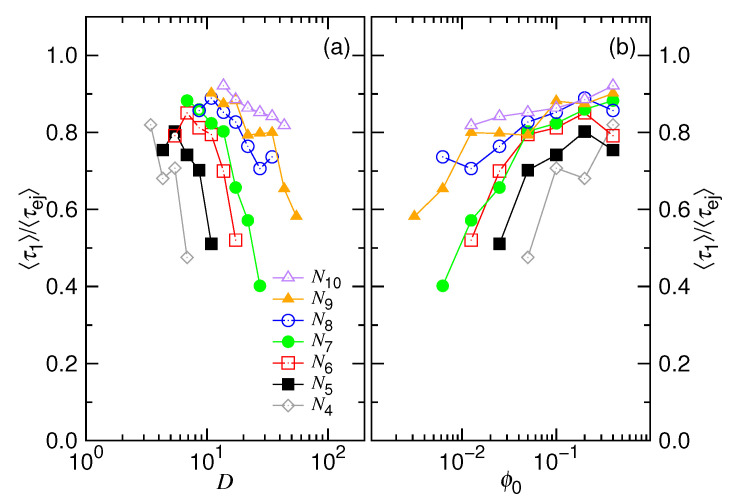
Ratio $\langle \tau_{1}\rangle /\langle \tau_{\mathrm{ej}}\rangle$ as a function of (**a**) *D* and (**b**) $\phi_{0}$. The chain length can be read in the legend of (**a**) where $N_{i}=2^{i}$.

We comment that there exist two situations where the nucleation will not occur prior to the ejection: either the osmotic pressure inside the cavity is higher than the one in the pore or the chain has been placed to traverse the pore channel since the beginning. For bacteriophages, both of the situations can help to skip or minimize the nucleation, because the packing fraction of DNA in the capsid is very high and the DNA chain could also have been hanged inside the tail tube of the virion before the ejection [1,2,58]. Concerning the dynamics, we do observe that the ejection slows down quite a lot for the major part of the process, because the internal pressure decreases, owing to the reduction of the number of monomers inside the cavity. However, a complete inhibition in the progress of ejection is not witnessed in our primitive model. From the view point of thermodynamics, the entropic force from the external portion of chain is stronger than the one from the internal. Consequently, the chain should be eventually retrieved out of the cavity. Hence, the rest of the process is accomplished by an acceleration of the ejection velocity. In order to understand the importance of the duration for the two stages, we calculate the ratio of the time at the confined stage to the total ejection time, $\langle \tau_{1}\rangle /\langle \tau_{\mathrm{ej}}\rangle$. The results are plotted in Figure 16 as a function of *D* and $\phi_{0}$ for different chain lengths.

We can see that $\langle \tau_{1}\rangle /\langle \tau_{\mathrm{ej}}\rangle$ decreases with increasing *D* or decreasing $\phi_{0}$. For the long chain case N=1024 with $\phi_{0}$ lying between 0.1 and 0.4 (a typical packing fraction for a bacteriophage), the ratio is around 0.9. It shows that about 90% of the ejection time is spent at the confined stage. The slow process at the second (non-confined) stage does not take a very long time, as imagined by some others. However, the inhibition of ejection for bacteriophages did happen in well-controlled experiments [11,13,14]. Therefore, some mechanisms beyond the scope of our model and thermodynamics must exist to cause the results.

The balance method that is used in this study can be derived under the framework of a general principle, called Onsager’s variational principle [59,60]. In it, a physical function, called Rayleighian, is established, which is the energy dissipation function plus the rate of change of the free energy. In our case, the Rayleighian is written as

(9) $$R\left(\dot{m};m\right)=\frac{1}{2}\eta \sigma^{2}{\dot{m}}^{2}+\frac{\partial F}{\partial m}\dot{m}$$

where the number of monomers in the cavity, *m*, is the state variable for describing the evolution of the ejection. The Rayleighian is regarded as a function of the time derivative of the state variable. The kinetic equation for *m* can be then determined by minimizing R with respect to $\dot{m}$. Thus, we have

(10) $$\frac{dm}{dt}=-\frac{1}{\eta \sigma^{2}}\frac{\partial F}{\partial m}$$

The same equation has been used in order to derive the dynamical equations in Equation (1) and Equation (4) for the confined and non-confined stages, respectively, as in Section 2. This principle is also called the principle of the least energy dissipation.

Concerning the confinement, the geometry of cavity is not always a sphere. For example, a common structure for virion is icosahedron [61,62]. Certain viruses, like T4 bacteriophages, have their icosahedral capsid elongated in the polar direction [63]. In addition to the icosahedral, helical nucleocapsid is also observed in nature, like in tobacco mosaic virus and Ebola virus; in this case, the morphology of virus is cylindrical [64,65]. In applications, biopolymers can be ejected from a confined space of any engineered shape, like an ellipsoid, a cylinder, a cone, or a slit. Equation (10) permits us to investigate these problems in a general way, provided that the free energy is known as a function of the state variable. Because the confining free energy of chain in an icosahedral container or an ellipsoid can be essentially described by the blob theory under the same form $F∼k_{B}Tm/g$, we expect that the ejection behavior maintains in a similar scaling class. The ejection exponents from an icosahedral or ellipsoidal cavity should be close to the ones from a spherical cavity.

In summary, we have developed a scaling theory in order to explain polymer ejection from a cavity in this study. The dynamical equation of ejection was derived by balancing the rate of the free energy change with the rate of the energy dissipation when the chain passes the pore. A two-stage model was used in order to describe the ejection process. At the confined stage, the chain suffers from the cavity confinement and it is pressed out of the cavity with a velocity $V_{\mathrm{ej}}∼\frac{\Delta v_{0}}{N^{x_{1}}}{\left(\frac{m}{{\left(D/\sigma \right)}^{3}}\right)}^{z_{1}}$. At the successive non-confined stage, the chain is driven by the entropic pulling from the external segments and the velocity scales as $m^{-z_{2}}\Delta v_{0}$. We have performed large-scaled molecular dynamics simulations to examine the velocity profile and confirmed that Equation (8) can describe the ejection velocity well with $z_{1}={\left(3\nu -1\right)}^{-1}$, $z_{2}=1+y_{2}$, $x_{1}=1/3$, and $y_{2}=2\nu -1$. The physical pictures for the origin of the four exponents have been clearly explained in the text. The exponent $z_{1}$ describes the decreasing behavior of the ejection velocity at the confined stage, while $z_{2}$ depicts the scaling increase of the velocity at the non-confined stage. $x_{1}$ is the exponent concerning the geometrical restriction and jamming that occurred at the pore when a chain is pressed out of the cavity and $y_{2}$ is the one accounting for the extra scaling dependence on *m* in the final phase of the process. The scaling properties of the threshold $m_{*}$, which separates the two stages, have also been investigated.

When studying the time evolution of 〈m〉 in the cavity, we observed that the number is stalled against decreasing for a long while. Detailed analysis revealed that a pre-stage exists for the heading monomers in order to find a way out of the pore and it is responsible for the stalling. By varying the pore length and the other simulation parameters, we have demonstrated that the pre-stage fulfills the characteristics of the Kramers escape problem and they can be thought as a nucleation phenomenon. After trimming the nucleation stage, the evolution of the monomer number in the cavity can be properly described by Equation (3) and Equation (7), and the fitting parameters were found to acquire the predicted exponents and scaling behaviors.

The total processing time 〈τ〉 was split into the ejection time $\langle \tau_{\mathrm{ej}}\rangle$ and nucleation time $\langle \tau_{n}\rangle$. Our simulations showed that $\langle \tau_{\mathrm{ej}}\rangle$ and $\langle \tau_{n}\rangle$ possess their own scaling. $\langle \tau_{\mathrm{ej}}\rangle$ scales as $N^{x_{1}}D^{2/\nu }$ when $N\geq N_{*}$ or $D\leq D_{*}$, and $N^{1+z_{2}}$ when $N<N_{*}$ or $D>D_{*}$. The previous scaling changes to $N^{x_{1}+\left(2/3\nu \right)}\phi_{0}^{-2/3\nu }$ if *D* is replaced by ${\left(N/\phi_{0}\right)}^{1/3}$, applied for the large *N* or large $\phi_{0}$ ($\phi_{0}\geq \phi_{*}$) case. If it is *N* being replaced by $\phi_{0}{\left(D/\sigma \right)}^{3}$, then the two scalings change to the forms $\phi_{0}^{x_{1}}D^{3x_{1}+\left(2/\nu \right)}$ and $\phi_{0}^{1+z_{2}}D^{3(1+z_{2})}$ for the large $\phi_{0}$ (or small *D*) and small $\phi_{0}$ (or large *D*) cases, respectively. For the nucleation time, we found that $\langle \tau_{n}\rangle ≃\frac{\sigma^{2}\eta }{k_{B}T}\exp\left(\frac{L_{p}\Delta \mu_{\mathrm{cp}}}{\sigma k_{B}T}\right)$ gives a good description. $\langle \tau_{n}\rangle$ increases with decreasing $\phi_{0}$ and the scaling changes from $N^{x_{1}}$ to $N^{1+\nu }$ when passing the demarcation line. A physical picture has been given, which connected the scaling variation with the change of the effective friction coefficient η. The picture also predicted the scaling $D^{3x_{1}}$ at large $\phi_{0}$ and $D^{3(1+\nu )}$ at small $\phi_{0}$. The simulations support fully the predictions, which are verified by stringent and various ejection conditions. It shows that the presented ejection theory is a consistent and complete theory for the primitive model. The results provide deep insight into the complex phenomena of ejection for biopolymers that occurred in nature and nanotechnology.

## Figures and Tables

**Table 1:** Ejection time $\tau_{\mathrm{ej}}$ expressed as a function of (a) *N* and *D*, (b) $\phi_{0}$ and *N*, and (c) *D* and $\phi_{0}$. Each expression comprises two pieces of function that are applied under different conditions. The right two columns in the table provide the two ways to regard $\tau_{\mathrm{ej}}$. For example, in the the right-most column for (a), the conditions ‘$D>D_{*}$’ and ‘$D\leq D_{*}$’ are given to separate $\tau_{\mathrm{ej}}$ into the two pieces as *N* is fixed. The definition of the critical value $D_{*}$ is given below.

	Ejection time as a function of *N* and *D*	if *D* is fixed	if *N* is fixed
(a)	$\frac{\tau_{\mathrm{ej}}\left(N,D\right)}{\Delta t_{0}}∼\left\{\begin{array}{c} \frac{A_{2}}{1+z_{2}}N^{1+z_{2}}\\ \frac{A_{1}N^{x_{1}}}{z_{1}-1}\left[{\left(\frac{D}{\sigma }\right)}^{\frac{2}{\nu }}-{\left(\frac{D}{\sigma }\right)}^{\frac{1+z_{1}}{\nu }}N^{1-z_{1}}\right]+\frac{A_{2}}{1+z_{2}}{\left(\frac{D}{\sigma }\right)}^{\frac{1+z_{2}}{\nu }} \end{array}\right.$	$\begin{array}{c} N<N_{*}\\ N\geq N_{*} \end{array}$	$\begin{array}{c} D>D_{*}\\ D\leq D_{*} \end{array}$
		$N_{*}∼{\left(\frac{D}{\sigma }\right)}^{\frac{1}{\nu }}$	$D_{*}∼\sigma N^{\nu }$
	Ejection time as a function of $\phi_{0}$ and *N*	if *N* is fixed	if $\phi_{0}$ is fixed
(b)	$\frac{\tau_{\mathrm{ej}}\left(\phi_{0},N\right)}{\Delta t_{0}}∼\left\{\begin{array}{c} \frac{A_{2}}{1+z_{2}}N^{1+z_{2}}\\ \frac{A_{1}N^{x_{1}}}{z_{1}-1}\left[{\left(\frac{N}{\phi_{0}}\right)}^{\frac{2}{3\nu }}-\frac{N}{\phi_{0}^{z_{1}}}\right]+\frac{A_{2}}{1+z_{2}}{\left(\frac{N}{\phi_{0}}\right)}^{\frac{1+z_{2}}{3\nu }} \end{array}\right.$	$\begin{array}{c} \phi_{0}<\phi_{*}\\ \phi_{0}\geq \phi_{*} \end{array}$	$\begin{array}{c} N<N_{*}\\ N\geq N_{*} \end{array}$
		$\phi_{*}∼N^{-\frac{1}{z_{1}}}$	$N_{*}∼\phi_{0}^{-z_{1}}$
	Ejection time as a function of *D* and $\phi_{0}$	if $\phi_{0}$ is fixed	if *D* is fixed
(c)	$\frac{\tau_{\mathrm{ej}}\left(D,\phi_{0}\right)}{\Delta t_{0}}∼\left\{\begin{array}{c} \frac{A_{2}}{1+z_{2}}{\left[\phi_{0}{\left(\frac{D}{\sigma }\right)}^{3}\right]}^{1+z_{2}}\\ \frac{A_{1}\phi_{0}^{x_{1}}}{z_{1}-1}{\left(\frac{D}{\sigma }\right)}^{3x_{1}}\left[{\left(\frac{D}{\sigma }\right)}^{\frac{2}{\nu }}-{\left(\frac{D}{\sigma }\right)}^{3}\phi_{0}^{1-z_{1}}\right]+\frac{A_{2}}{1+z_{2}}{\left(\frac{D}{\sigma }\right)}^{\frac{1+z_{2}}{\nu }} \end{array}\right.$	$\begin{array}{c} D>D_{*}\\ D\leq D_{*} \end{array}$	$\begin{array}{c} \phi_{0}<\phi_{*}\\ \phi_{0}\geq \phi_{*} \end{array}$
		$D_{*}∼\sigma \phi_{0}^{-\nu z_{1}}$	$\phi_{*}∼{\left(\frac{D}{\sigma }\right)}^{-\frac{1}{\nu z_{1}}}$

## Supplementary Materials

The following are available online at https://www.mdpi.com/2073-4360/12/12/3014/s1, Figure S1: $m_{*}$ versus *N* under (a) the $\phi_{0}$-fixed condition and (b) the *D*-fixed condition.
