# Supplementary material for: Scaling Theory of a Polymer Ejecting from a Cavity into a Semi-Space
Source: Polymers (Basel). 2020 Dec 16;12(12):3014. doi: 10.3390/polym12123014 (PMC7766115; doi:10.3390/polym12123014)
Supplement: Supplementary file 1 [file polymers-12-03014-s001.pdf]

Article

Supporting Information: Scaling Theory of a Polymer Ejecting from a Cavity into a Semi-Space

Pai-Yi Hsiao [1,2,*]

[1] Department of Engineering and System Science, National Tsing Hua University, Hsinchu, Taiwan, R. O. C.
[2] Institute of Nuclear Engineering and Science, National Tsing Hua University, Hsinchu, Taiwan, R. O. C.
Email: pyhsiao@ess.nthu.edu.tw; pyhsiao@mx.nthu.edu.tw

Version November 18, 2020 submitted to Journal Not Specified

S1. Critical monomer number m_* versus N

Figure S1 presents the variation of the critical monomer number m_* as a function of N. We recall that m_* separates the confined and the non-confined stage in an ejection process and is determined by finding the location of the minimum in an ejection velocity. Panel (a) shows that the m_* curve increases with $N^{0.58(2)}$ at a given ϕ_0. The behavior agrees with the theoretical prediction $m_* \sim (N/\phi_0)^{1/(3\nu)}$ by setting $\nu = 0.6$. A larger ϕ_0 value leads to a smaller m_*. As a result, the m_* curves are appeared in parallel for different ϕ_0 in the plot. If D is fixed, a null dependence on N is predicted because of $m_* \sim (D/\sigma)^{1/\nu}$. It is consistent with our result shown in Panel (b).

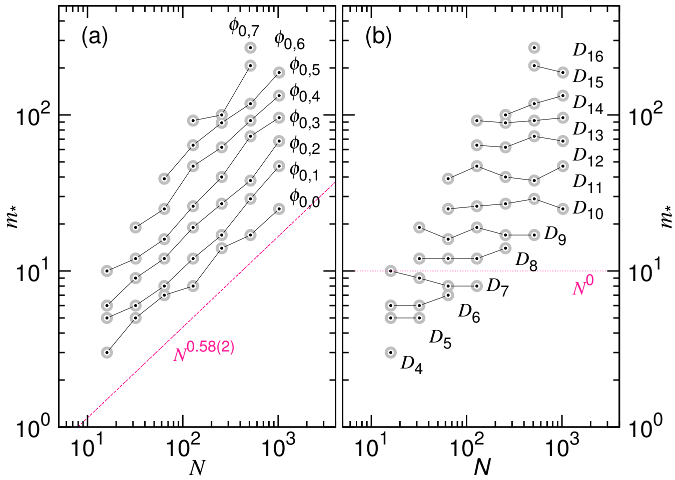

Figure S1. Critical monomer number m_* as a function of the chain length N under (a) the ϕ_0-fixed condition and (b) the D-fixed condition. The values of ϕ_0 and D are indicated near the corresponding curves where $\phi_{0,g} = 0.4 \times 2^{-g}$ and $D_j = \sqrt[3]{2.5 \times 2^j}$.

Publisher's Note: MDPI stays neutral with regard to jurisdictional claims in published maps and institutional affiliations.

© 2020 by the author. Submitted to *Journal Not Specified* for possible open access publication under the terms and conditions of the Creative Commons Attribution (CC BY) license (http://creativecommons.org/licenses/by/4.0/).
